# Exploring Plants with Flowers: From Therapeutic Nutritional Benefits to Innovative Sustainable Uses

**DOI:** 10.3390/foods12224066

**Published:** 2023-11-08

**Authors:** Elena Coyago-Cruz, Melany Moya, Gabriela Méndez, Michael Villacís, Patricio Rojas-Silva, Mireia Corell, Paula Mapelli-Brahm, Isabel M. Vicario, Antonio J. Meléndez-Martínez

**Affiliations:** 1 Carrera de Ingeniería en Biotecnología de los Recursos Naturales, Universidad Politécnica Salesiana, Sede Quito, Campus El Girón, Av. 12 de Octubre N2422 y Wilson, Quito 170143, Ecuador; 2 Facultad de Ciencias Médicas, Carrera de Obstetricia, Universidad Central del Ecuador, Iquique, Luis Sodiro N14-121, Quito 170146, Ecuador; 3 Instituto de Microbiología, Colegio de Ciencias Biológicas y Ambientales COCIBA, Universidad San Francisco de Quito USFQ, Quito 170901, Ecuador; 4 Departamento de Ciencias Agroforestales, Escuela Técnica Superior de Ingeniería Agronómica, Universidad de Sevilla, Carretera de Utrera Km 1, 41013 Sevilla, Spain; 5 Unidad Asociada al CSIC de Uso Sostenible del Suelo y el Agua en la Agricultura (US-IRNAS), Crta. de Utrera Km 1, 41013 Sevilla, Spain; 6 Food Colour and Quality Laboratory, Facultad de Farmacia, Universidad de Sevilla, 41012 Sevilla, Spainajmelendez@us.es (A.J.M.-M.)

**Keywords:** carotenoids, edible flowers, flavonoids, functional foods, nutraceuticals, phenolic compounds, natural dyes

## Abstract

Flowers have played a significant role in society, focusing on their aesthetic value rather than their food potential. This study’s goal was to look into flowering plants for everything from health benefits to other possible applications. This review presents detailed information on 119 species of flowers with agri-food and health relevance. Data were collected on their family, species, common name, commonly used plant part, bioremediation applications, main chemical compounds, medicinal and gastronomic uses, and concentration of bioactive compounds such as carotenoids and phenolic compounds. In this respect, 87% of the floral species studied contain some toxic compounds, sometimes making them inedible, but specific molecules from these species have been used in medicine. Seventy-six percent can be consumed in low doses by infusion. In addition, 97% of the species studied are reported to have medicinal uses (32% immune system), and 63% could be used in the bioremediation of contaminated environments. Significantly, more than 50% of the species were only analysed for total concentrations of carotenoids and phenolic compounds, indicating a significant gap in identifying specific molecules of these bioactive compounds. These potential sources of bioactive compounds could transform the health and nutraceutical industries, offering innovative approaches to combat oxidative stress and promote optimal well-being.

## 1. Introduction

Since time immemorial, flowers have played a fundamental role in society. They are appreciated for their beauty and used for ornamental purposes in various spaces, whether in pots, gardens, landscaping, or as cut flower arrangements in containers. Grown specifically for their striking appearance, distinctive foliage, and delicate fragrance, ornamental plants add charm and distinction to any environment. Their presence goes beyond mere aesthetics, as throughout history, flowers have been symbols of emotion, used in celebrations, expressions of love, condolences, and religious rituals, and providing benefits for emotional well-being and mental health [1].

In recent years, the market for edible flowers has experienced remarkable growth. This phenomenon can be attributed to several reasons, including the increasing availability of information on their nutritional value and bioactive potential [2,3]. In addition, there has been a growing interest in the potential health benefits of specific secondary metabolites and other compounds commonly found in flowers, such as carotenoids, phenolic compounds, vitamins C and E, saponins, or phytosterols [4]. Carotenoids and phenolic compounds are responsible for the different colours of flowers [5,6] stand out for their health properties and versatility in agri-food and health applications [7,8,9,10].

Flowers are used in gastronomy for their pigments (such as carotenoids, flavonoids, and betalains), which improve the appearance of dishes [11], and for their characteristic flavours and odours, which make them an alternative food source [12]. Some societies, such as Asian, Greek, Ancient Roman, French, and Italian, have a long-standing tradition of eating flowers [3]. However, for a flower to be edible, it must not contain dangerous levels of toxic compounds that could affect the health of those who consume it [13,14]. On the other hand, the consumption of parts of plants is typical in traditional medicine, mainly in medicinal infusions or decoctions [15,16]. Flowers are recognised as alternative food sources to improve health and contribute to food security [4]. As sustainability is a global priority, especially in food production, which is considered the most significant human pressure on the Earth [17], using cultivated flowers for gastronomic purposes can be aligned with a responsible approach towards the environment and general well-being.

This review aimed to collect relevant information on ornamental plant flowers with potential health promotion as botanicals, foods, or other uses, following sustainability principles and the circular economy. Plants from fifty families are covered, including Asteraceae, Lamiaceae, Fabaceae, and Malvaceae, as well as plants with edible flowers from the families Asteraceae, Apiaceae, Brassicaceae, Oleacaceae, Malvaceae, and Ranunculaceae. Therefore, Table 1 contains a compilation of common plants characterised by their flowers, with detailed information on the family, common name, place of origin, part of the commonly used plant, uses in bioremediation, main chemical compounds, medicinal uses, gastronomic uses, and concentration of carotenoids and phenolic compounds, together with the technique used for each flower species. In addition, data on carotenoids and phenolic compounds in different flower species are presented in Table 2 and Table 3, respectively. This review aims to provide a comprehensive overview of flower possibilities and benefits in other areas, highlighting their potential in harmony with nature and general well-being.

## 2. Conceptualization

### 2.1. Use of Flowers for Therapeutic and Nutritional Purposes throughout History

The use of flowers in food and medicine has a long history dating back to antiquity. The earliest records of these uses date back to 4000 BC in Mesopotamian and Egyptian cultures [439]. An emblematic example of the traditional use of a flower for medicinal purposes is chamomile (*Chamomilla recutita* L.), whose dried flowers have been used since ancient times to treat menstrual disorders, insomnia, ulcers, haemorrhoids, and other ailments [440]. Similarly, cannabis (*Cannabis sativa* L.), known for its soothing and anticonvulsant properties, has been used therapeutically in many cultures. This plant was even included in the British and later American pharmacopoeia but was eliminated in the 20th century because of concerns about the risk of abuse and intoxication [441]. Similarly, flowers of the Asteraceae family (*Achillea millefolium* L., *Arnica montana* L., *Bellis perennis* L., *Calendula officinalis* L., *Chamaemelum nobile* (L.) All., *Helichrysum stoechas* (L.) Moench, and *Taraxacum officinale* L.) have been traditionally used in folk medicine for various therapeutic purposes [442].

Around 180 plant species belonging to 97 families have been reported as producing edible flowers [3]. The use of flowers in gastronomy is documented in various cultures, such as Roman, Greek, Chinese, Indian, and Central European [443,444], where they are used to enhance both the presentation and nutritional value of food. In ancient Rome, several roses were often used in omelettes and purées. In mediaeval France, marigolds were a common ingredient in salads. The stigmas of the saffron flower are one of the most commonly used colouring and flavouring agents in cooking. Similarly, violet petals were used to make sweets and colour sugar, while dandelion flowers were used in drinks and salads [12]. Furthermore, in the Mediterranean diet, some commonly consumed vegetables are flowers, such as artichokes, capers, broccoli, or cauliflower. Worldwide, edible flowers such as pansy, marigold, borage, nasturtium, mini rose, torenia, mini daisy, cosmos, clitoria, craving, begonia, sunflower, snapdragon, and squash blossom are appreciated and consumed in different countries such as Brazil, the United States, France, Italy, Portugal, China, and Japan [4].

**Figure 1:**
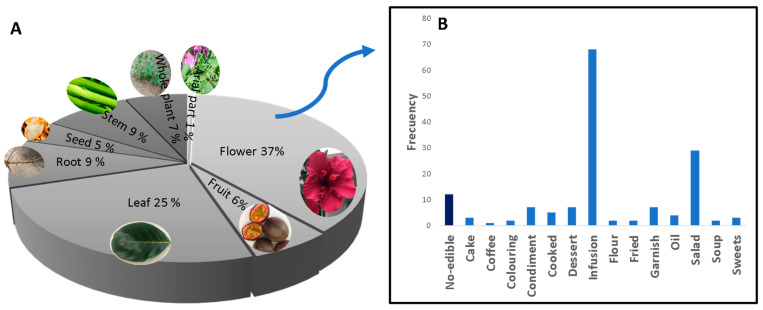
Parts of the plant used in food (**A**) and frequency of use in different dishes (**B**).

Nowadays, thanks mainly to globalisation, the use of flowers in gastronomy is growing steadily (Figure 1). The inclusion of flowers in the culinary creations of renowned innovative chefs has attracted great attention in this field, leading to the emergence of companies specialising in producing flowers for this booming market [445]. Although flowers are now widely used as condiments, decorative elements, or for flavouring dishes, their potential as a source of nutrients or other valuable nutritional compounds must be further explored [446]. It is important to note that although there are several reviews documenting the use of many flowers as food or for therapeutic purposes [12,444,447]. To date, no official list of edible flowers has been developed, and no specific legislation on their use and applicability has been established by international bodies such as the FAO, WHO, FDA, or EFSA. As interest in the culinary use of flowers grows, an informed and responsible approach is needed to ensure their safety and sustainably exploit their nutritional and medicinal potential.

### 2.2. Post-Harvest Treatments Applied to Flowers

Thanks to the undoubted increase in demand for edible flowers, there is a growing body of research on how to extend their shelf life and improve their overall quality by enhancing post-harvest treatments [2,448,449]. This could benefit our health and improve their industrial development [2]. However, nowadays, despite their short life (early petal abscission and discoloration, flower wilt, dehydration, and tissue browning), edible flowers are usually sold fresh and chilled without any other postharvest treatment. In addition to refrigeration, other common post-harvest methods include crystallisation, freeze-drying, sugar canning, and preservation in distillates [4,450].

Several new food preservation technologies have been investigated to increase the shelf life of edible flowers. Among them are high hydrostatic pressure (HHP), irradiation, ultraviolet, ionising radiation, and new packaging alternatives [2,4]. On the other hand, other studies have evaluated how post-harvest treatments can affect bioactive compounds present in edible plants. For example, it has been shown that freeze-drying decreases the loss of carotenoids (in daylilies and marigolds), caffeic acid derivatives, and total phenolics (in purple coneflower) compared to hot-air drying [451,452,453].

### 2.3. Forms of Consumption

Edible flowers are mainly used fresh as decoration or a garnish for some meals, such as salads or light curry, adding colour and fragrance. In addition, they are also used for other culinary purposes, such as ingredients in bread, pancakes, sauces, jellies, syrups, vinegar, honey, oils, soups, infusions, flower-scented sugars, candied flowers, cheeses, ice cream, crisps, juices, rice, cakes, butter, pasta, wine, and flavoured liqueurs. Edible flowers can even be consumed dried, in ice cubes in cocktails, directly as vegetables, or in stir-fried dishes [3,4,12,454]. The flowers are typically eaten whole, but there are some species of which only some parts are adequate for consumption. For example, some parts of flowers are too bitter, such as the white parts of the roses and the base of the chrysanthemum petals, or too rough, such as some parts of the blueweed (*Echium vulgare* L.). Other examples of flowers that are not eaten whole include tulips (*Tulipa* spp.) and chrysanthemums (*Chrysanthemum*) (only the petals are consumed), daisies (*Bellis perennis* L.) and garden nasturtium (*Tropaeolum majus* L.) (only the flower buds), and pumpkins (*Cucurbita* spp.) (only the tiny and undeveloped fruits with flowers) [12,444]. Information about the use of flowers for food is summarised in Table 1.

### 2.4. Relevant Compounds with an Interest in Nutrition, Health Promotion, and Cosmetics

Using flowers as food is not exclusively for aesthetic reasons; the nutritional contribution should also be considered. Flowers provide important elements for nutrition and health. Some flowers contain proteins, fats, carbohydrates, vitamins A, B, C, and E, mineral elements, and bioactives [447]. The concentration of minerals in flowers is such that, taking into account the Dietary Reference Intakes for an adult for magnesium (375 mg/day), phosphorus (700 mg/day), and potassium (2000 mg/day), it can be concluded that the consumption of some edible flowers could help to meet these daily requirements [444], although the boiling process of some flowers significantly reduces the mineral concentration [455].

The presence of compounds with nutritional interest differs across floral structures. Pollen has high concentrations of proteins, amino acids, carbohydrates, and lipids, among other nutrients. Nevertheless, the amount of pollen in the flower is very small, and in addition, it has a flat taste without individual characteristics. Nectar, which typically has a sweet taste, contains a balanced mixture of sugars (fructose, glucose, and sucrose), amino acids (mainly prolin), proteins, inorganic ions, lipids, organic acids, alkaloids, etc. Lastly, the petals and the rest of the flower may also be significant sources of vitamins, minerals, and bioactive compounds [12,444].

**Figure 2:**
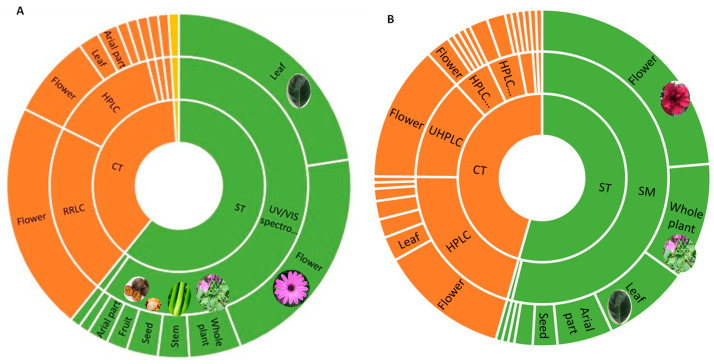
Study the distribution of carotenoids (**A**) and phenolic compounds (**B**) in different parts of the plant using spectrophotometric (ST) and chromatographic techniques (CT).

Although before 2000, studies on edible flowers focused mainly on their nutrients, recent research has revealed the importance of studying compounds with bioactive properties. Phenolic compounds (flavonols, flavones, anthocyanins, and phenolic acids) and carotenoids are among the main bioactive compounds. The concentration of carotenoids and phenolic compounds in plants is detailed in Table 2, while the techniques used for quantification are shown in Figure 2. For both carotenoids (60.8%) and phenolics (54.4%), spectrophotometric techniques have been used. In the case of carotenoids, the focus has been on the study of leaves, while phenols have been studied in flowers. In addition, the chromatographic techniques used for carotenoids were mainly RRLC (55.0%) and HPLC (35%), while for phenols, the main techniques were HPLC (44.9%) and UHPLC (28.6%). In addition, information about carotenoids and phenolic compounds present in flowers is summarised in Table 3 and Table 4, respectively.

These compounds usually account for their colour, either directly or indirectly through copigmentation [3,6,447]. In a recent study in which 125 flower species (of which 111 were edible) were surveyed for their colour (white, yellow, orange, pink, red, lilac, and blue), carotenoids, and phenolic compounds, it was observed that overall, flowers with high carotenoid contents did not contain high phenolic contents and vice versa [366]. Quercetin, kaempferol, isorhamnetin, myricetin, and their derivatives have been reported to be significant flavonols in flowers and represent their main class of flavonoids. The second major class of flavonoids in edible flowers is that of flavones, such as luteolin, apigenin, acacetin, and chrysoeriol. Among the anthocyanins, the most common in flowers are pelargonidin, cyanidin, and delphinidin. The phenolic acids in edible flowers include chlorogenic acid, caffeic acid, caffeoylquinic acid, protocatechuic acid, and gallic acid (Table 4). Lastly, carotenoids are also common in flowers, mainly hydroxy xanthophylls, such as lutein, β-cryptoxanthin, and zeaxanthin, and xanthophylls containing hydroxyl and epoxide groups, such as violaxanthin, anteraxanthin, neoxanthin, and lutein-5,6-epoxide (Table 3). Provitamin A carotenes, such as α- and β-carotene, can also be found in flowers, as well as the colourless carotene phytoene [366], which has been largely neglected together with the colourless phytofluene in food science and nutrition but is attracting increasing attention [461]. Extraordinary high levels of the provitamin A carotenoids α-(1451.9 µg/g DW) and β-carotene (1362.2 µg/g DW) have recently been reported in *Renealmia alpinia* (Rottb.) Maas [366].

**Figure 3:**
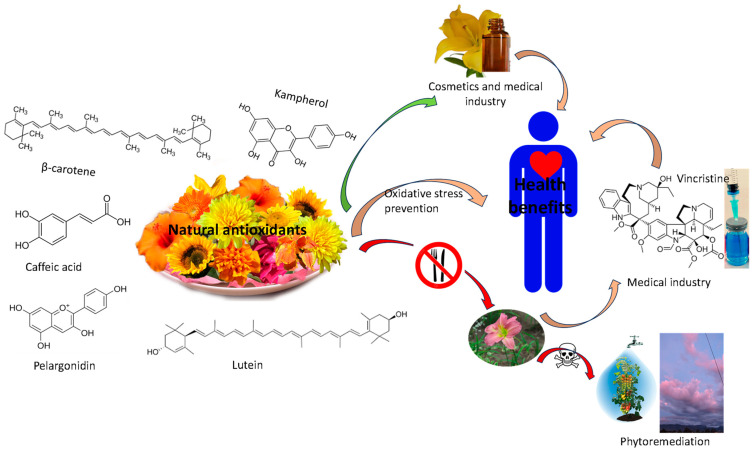
Potential uses of flowers.

Both phenolic compounds and carotenoids have been attracting a great deal of interest in recent decades about their possible health-promoting biological actions [462], hence their interest in the development of innovative products for health or well-being, including nutricosmetics [8,463,464] (Figure 3).

Phytosterols (β-sitosterol), alkaloids, lignans, neolignanes, coumarins, and bisabolol oxides A and B are other phytochemicals distributed in edible flowers. However, these compounds are usually present in smaller concentrations [3,447].

### 2.5. Beneficial Effects

**Figure 4:**
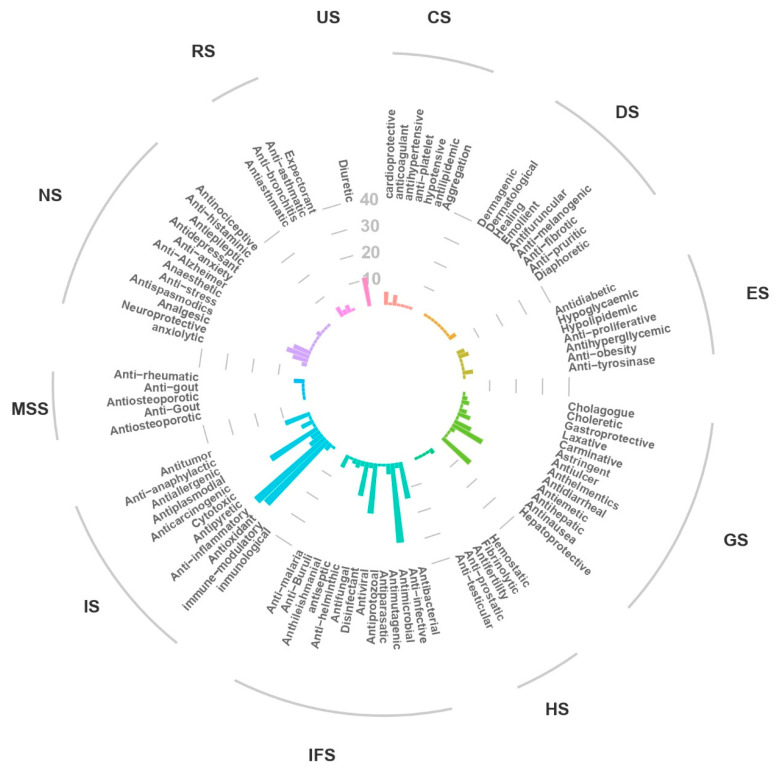
Plant species have activities beneficial to human health. Note: NS, Nervous system; RS, Respiratory system; US, Urinary system; CS, Cardiovascular system; (DS), Dermatological system; ES, Endocrine system; GS, Gastrointestinal system; HS, Haematological system; IFS, Infectious system; IS, Immune system; MSS, Musculoskeletal system.

Among the beneficial actions attributed to various flowers are antioxidants, anti-inflammatory, anti-carcinogenic, anti-obesity, hepatoprotective, neuroprotective, gastroprotective, antidiarrheal, anti-infective, antitumor, antispasmodic, analgesic, and astringent, among others [3,12,465] (Figure 4). However, studies have focused on the benefits these species can provide to the immune (31.5%), infectious (26.0%), and gastrointestinal (14.2%) systems. The flowers of begonias, roses, garden nasturtiums, daylily, calendula, Japanese rose, Daurian rose, daylily, and chrysanthemum might protect against oxidation, as there are studies indicating that they exhibit antioxidant capacity in vitro [6,12,447,466].

Some studies indicate that certain edible flowers can exhibit anti-carcinogenic activity against liver, colon, brain, skin, bladder, prostate, or breast cancers [3,12,444]. Examples are flowers from hibiscus, rose, chrysanthemum, tagetes, cosmos (*Cosmos sulphureus* Cav.), coral vine (*Antigonon leptopus* Hook. & Arn.), lesser bougainvillea (*Bougainvillea glabra* Choisy), jasmine, honeysuckle rose, cassia fistula, chives, calendula, and pomegranate [3,6,444,447,467].

Several flowers may exhibit anti-inflammatory activity. Examples are Roselle, Hangzhou white chrysanthemum, wild chrysanthemum, honeysuckle, and daylily. Antiobesity effects have been observed in flowers such as Roselle, magnolia, and waterlily. According to the literature, other beneficial effects that can be derived from the consumption of flowers include neuroprotective, visceral injury prevention, anti-diabetic, and antimicrobial effects, among others [3,12,110,444,468]. Edible flowers have also been suggested as fibre food sources, which may be attractive for developing dietary supplements for athletes [468].

Others, such as *Hibiscus rosa-sinensis* L., *Chrysanthemum* spp., *Dahlia coccinea* Cav., and *Citrullus lanatus* (Thunb.) Matsum. & Nakai, could protect against diseases linked to obesity (such as, for example, sleep apnea, hypertension, hyperlipidaemia and type 2 diabetes), neurological (Alzheimer and Parkinson) [469] and liver and gastrointestinal disorders [435].

Finally, the carotenoids lutein and zeaxanthin, present in high concentrations in the petals of the tagete flowers (*Tagetes erecta* L.), can act as a filter that protects the macula from blue light and oxidative damage. Thus, various studies have suggested that they could reduce the risk of ocular pathologies, especially age-related macular degeneration [447]. These carotenoids also attract attention as they could be involved in cognitive benefits [470]. Information about the medical uses of flowers is summarised in Table 1.

### 2.6. Antinutrients or Toxic Compounds

Although there is a wide variety of edible flowers, care must be taken since some flowers are poisonous or contain antinutrients. In any case, it is essential to consider that there are some techniques that people have learned over the years to eliminate or diminish antinutrients and toxic compounds. For example, people reduce the toxicity of flowers from *Erythrina* species (4.9 and 6.3 trypsin inhibitors/mg sample) caused by alkaloids by cooking the flowers and eliminating the cooking water [455].

Regarding the possible effects of the toxic flowers, these can vary from minor effects on the skin (such as skin allergies, dermatitis, or skin lesions) to death when ingested. The toxicity of plants is mainly due to compounds such as alkaloids, tannins, alcohols, phytotoxins, glycosides, resins, nitrites, photosensitising substances, and calcium oxalates. Toxic compounds may be present in plants naturally or due to environmental pollution (pesticides, heavy metals, hydrocarbons, etc.), living agents, or diseases [447]. Examples of natural toxicity in flowers are the Adonis flower (*Adonis aestivalis* L.), which contains cardioactive steroids resembling digitalis, or Chrysanthemum (*Chrysanthemum* species), which contains sesquiterpene lactones [471]. Information on the toxicity of plants can be found in European Regulation EC No. 258/97. Examples of induced toxicity in flowers are *Amaranthus hybridus* (500.0 mg Pb/kg plant) and *Medicago sativa* L. (720.0 mg Pb/kg plant), species used for phytoremediation processes (recovery of heavy metals and other pollutants) [14]. On the other hand, since flowers are often consumed fresh or minimally processed, they can pose microbiological risks [472].

Antinutrients are substances that have a negative impact on our nutrition by preventing the absorption or assimilation of a nutrient or inactivating its effect [473]. Some antinutrients found in flowers are tannins, phytic acid, oxalate, lectins, and saponins. Tannins are phenolic compounds that inhibit the metabolism of digested and absorbed proteins. The flowers of the genus *Rosa* usually contain high levels of tannins, such as gallotannins and ellagitannins. The flowers of *Woodfordia fruticosa* Salisb. (0.2 g/100 g DW) and *Ensete superbum* (Roxb.) Cheesman (0.003 g/100 g DW) also have tannins [466,473] Phytic acid, which is present, for example, in *E. superbum* (0.1 g/100 g DW), decreases the bioavailability of some minerals and proteins, but its content can be reduced by processing [473]. On the other hand, oxalate hinders calcium absorption and stimulates the formation of kidney stones. Although in low concentration, oxalate is present in edible flowers such as *Parkia biglobosa*, usually consumed in Nigeria; *E. superbum* (0.03 g/100 g DW); and *Woodfordia fruticosa* (0.06 g/100 g DW) [473,474]. Lectins, a major family of protein antinutrients, are found, for example, in the flower of *A. xalapensis* [455]. Saponins reduce glucose and cholesterol uptake, among other nutrients [473], and are present in the flowers of *A. salmiana* and *Y. filifera* [455]. In any case, it is important to note that compounds traditionally considered antinutrients could also exert beneficial effects. For example, under certain conditions, reducing the absorption of nutrients such as glucose or specific lipids could be desirable [475]. Information about toxic compounds in flowers is summarised in Table 1.

### 2.7. Flowers, Climate Change, Sustainability, and the Circular Economy

Flowers can become alternative sources of health-promoting dietary sources and sustainably contribute to food security, as suggested in an insightful review that addresses important topics of the 17 goals of the United Nations to transform our world included in the 2030 Agenda for Sustainable Development [4]. The agri-food industry is responsible for the biggest pressure caused by humans on Earth, especially due to its global freshwater land usage and contribution to greenhouse gas emissions. Paradoxically, 820 million people have insufficient food, and many more have unhealthy dietary patterns that considerably increase the risk of developing diseases and premature death. In this sense, global scientific goals towards a worldwide transformation of the agri-food system, aligned with global agendas, including the United Nations Sustainable Development Goals and the Paris Agreement, are being advocated [17]. In this scenario, the classical ‘take-make-consume and dispose’ linear economy model needs to be replaced by a circular economy model that ‘keeps the added value in products for as long as possible and eliminates waste’ [476].

#### 2.7.1. Flowers and Biodiversity

Taking advantage of biodiversity (the variety of life at the genetic, species, and ecosystem levels) is very important in this scenario. Biodiversity for food and agriculture is a subset that contributes directly and indirectly to agri-food. It encompasses “domesticated plants and animals raised in crop, livestock, forest, and aquaculture systems; harvested forest and aquatic species; wild relatives of domesticated species; other wild species harvested for food and other products; and what is known as ‘associated biodiversity’, the wide range of organisms that live in and around food and agricultural production systems, sustaining them and contributing to their production. Agriculture is considered here to include crop and livestock production, forestry, fisheries, and aquaculture. The importance of biodiversity in this context is at different levels, as many untapped edible species are very nutritious, are adapted to diverse edaphoclimatic conditions, and provide important ecosystem services [477]. Flowers play a relevant role in maintaining and promoting biodiversity, acting as pollen sources visited by bees and other social insects [4].

#### 2.7.2. Flowers and Phytoremediation of Soils and Wastewater

Food production is estimated to account for ~40% of land and 70% of freshwater use, which are precious and increasingly scarce resources. Therefore, contamination of soil and water is an important problem. More specifically, the elevated presence of heavy metals, other minerals, or organic pollutants in soils used for food production is an important environmental problem, a great threat to life on earth, and poses health risks when they enter the food chain. Being non-biodegradable, heavy metals accumulate in the environment and enter the food chain. This is undesirable from environmental and health standpoints, as some heavy metals are carcinogenic, mutagenic, teratogenic, and endocrine disruptors, while others cause neurological and behavioural changes. Naturally present or derived from anthropogenic sources, such metals can be reduced using physical and chemical approaches. However, these are costly and laborious and can lead to disturbances in physicochemical and microbial soil characteristics. Phytoremediation is gaining importance due to its public acceptance, efficiency, cost-effectiveness, and eco-friendliness. It can also reduce organic pollutants such as polynuclear aromatic hydrocarbons, polychlorinated biphenyls, and pesticides [478].

It uses green plants and associated microbes to minimise the toxic effects of potential contaminants in the environment. There are several ways to remediate. Phytostabilisation or phytoimmobilisation refers to the decrease in the mobility or/and bioavailability of a metal, which impairs its leaching to water or its entry into the food chain. Once uptaken by roots, a specific heavy metal may either be phytoimmobilised there or translocated to aerial parts. Phytovolatilisation involves the conversion of the metal into a volatile form and its release into the atmosphere through stomata. This technique is primarily helpful for Hg, although the volatilised metal can eventually return to the soil through precipitation. Phytodegradation is the degradation of organic pollutants by plants with the help of enzymes. Rhizodegradation refers to the breakdown of organic contaminants in the soil by microorganisms in the rhizosphere. Phytodesalination refers to the use of halophytic plants for the removal of salts from salt-affected soils to enable them to support normal plant growth [478,479]. Plants are categorised based on their metal uptake mechanisms: excluders restrict heavy metal uptake and accumulation to the shoot; indicators/accumulators accumulate them in aerial parts comparatively the same as the soil levels; and hyperaccumulators uptake and translocate the metals to shoots and leaves without toxic symptoms. Different detoxification strategies include compartmentalisation, deposition, distribution, and stabilisation within cell walls, vacuoles, and metabolically inactive tissues. Plants of great value in floriculture from the Asteraceae family (for instance, *Tagetes erecta*, *Calendula officinalis*, and *Chrysanthemum indicum*) have been reported to tolerate heavy metal soil pollution [480].

Phytoextraction is considered the most important phytoremediation approach and can be more suitable for commercial applications. Ideally, plants selected for phytoextraction should be widely distributed, easy to cultivate, rapidly grow, produce important amounts of biomass, and be poly-harvest. About metals, they should be hyperaccumulators of heavy metals, translocate them from root to shoot, and tolerate their toxic effects well. Additionally, plants should be resistant to biotic stresses, well adapted to edaphoclimatic conditions, and not attractive to herbivores to avoid the entry of heavy metals into the food chain. Although promising, phytoremediation has yet to be widely used at a large scale due to limitations of diverse nature (slow-growing species, low bioavailability of the metals, and long times to achieve decontamination, among others). New approaches are being evaluated, including assistance with chelators, biochar, bacteria, fungi, microbes, and transgenic plants [479,480].

Plant biomass enriched with phytoextracted heavy metals can be incinerated for energy and ash recovery. The latter can be considered bio-ore and be further processed for extracting heavy metals, a process called phytomining [478,480].

Phytoremediation has also been proven to be cost-effective and technically feasible in the remediation of heavy metal pollution in water quality issues, including wastewater treatment [478,480]. Reusing cut flowers and floral waste as a neat bio-adsorbent and activated carbon for removing the antibiotic levofloxacin and lead ions from water with promising results has been recently described [481].

As can be seen in Table 1, there are many reported uses of plants with ornamental flowers in the phytoremediation of various pollutants (heavy metals, radioactive elements, polycyclic aromatics and other hydrocarbons, benzene, textile dyes, oils, fertilisers, carbamazepine, insecticides, herbicides, and dioxins), which widens the use of such plants beyond ornamental, nutritional, or culinary purposes, which is an advantage in a circular economy model where the use of resources for several applications is desired. Interestingly, they can be found in locations with marked edaphoclimatic conditions, including arctic regions (Canada, Siberia), temperate regions (Mediterranean Basin, Central and Eastern Europe), or subtropical or tropical regions (Caribbean Basin) (Table 1).

Although the uptake of heavy metals and other pollutants could sometimes affect the use of flowers for human consumption (in those cases where contaminants are transferred to aerial plants from the roots and they are rich in unsafe concentrations), others are feasible, including pot plants, cut flowers, essential oils, perfumes, air freshener production, metal phytomining, and feedstock in silk production [480].

#### 2.7.3. Flowers and Dying Fabrics

The replacement of synthetic dyes with more natural alternatives in textiles is gaining acceptance to reduce the negative environmental impacts and toxicity associated with the latter. Besides, some natural extracts can exhibit properties (antioxidative, antimicrobial, UV-light absorption, etc.), which can be interesting for developing functional fabrics with added value. The application of natural dyes in the textile industry is gaining popularity due to the increasing awareness of the environmental, ecological, and pollution caused by synthetic dyes. Different conditions of pH, temperature, salt, time, chemical levels, and biomordants have been tested for the dying of wool with rose flowers. As a result, different colour hues were obtained, some with good colour strength. [482]. *Kigelia africana* flowers have also been studied as possible materials for the functional colouration of textile materials, with promising results in terms of fastness, colour strength, antibacterial, antioxidant, and UV-protection properties [483].

#### 2.7.4. Other Potential Uses

Waste jasmine flowers have recently been tested to produce bioethanol mediated by immobilised yeasts. Pretreatments that included alkalinisation, heating, and enzymatic hydrolysis were evaluated to favour the accessibility of the carbohydrate fraction, and response surface methodology was applied to assess the interactions of different variables to better understand the bioethanol yield [484].

Saffron purple petals have been evaluated as a possible environmental-friendly additive for bentonite-based drilling fluids, and significant enhanced rheological, filtration, and corrosion protection properties were observed.

Drilling fluids are circulated in boreholes to help perform an efficient drilling operation with minimal damage to prospective formations [485].

Porous carbon nanosheets have been obtained from the carbonisation of a paper flower. The materials exhibited interesting properties for their potential use in energy storage and dye removal [486].

## 3. Conclusions and Future Recommendations

Flowers have played a fundamental role in human culture over the centuries, finding value in both their aesthetic appeal and their nutritional and therapeutic properties. A vivid example of this duality is theprized *Tagetes erecta*, whose flowers are commercially exploited both for their exquisite ornamental beauty and for their remarkable lutein content, a valuable carotenoids widely used in industry as a dye and as a key component of health-promoting dietary supplements. In a similar context, the flowers of *Renealmia alpinia* have emerged as botanical gems, standing out as rich sources of provitamin A carotenoids such as α- and β-carotene [9,366]. This discovery has led to growing interest both in the culinary field, where they are used to enrich dishes, and in the search for health-promoting bioactive compounds [3,6,444]. However, it is important to recognise that while flowers offer a wealth of benefits, some of them contain potentially toxic compounds, which means that their use in gastronomy and therapy must be approached with caution. One particularly promising aspect is the medicinal potential of a group of 115 species of flowers, all of which have properties that suggest their usefulness in the treatment of various conditions. This opens an exciting field of research where new molecules may be discovered.

However, they can also be used for other purposes at different points in their life cycles, including as phytoremediators [480], dying agents for textiles [483], or feedstocks for bioethanol production, among others [484]. Although recent research on these topics is promising to pave the way for the circular use of flowers to produce sustainable and health-promoting foods, there is still a long way to go. Of course, lifecycle assessments must be performed, and more scientific evidence is required to bridge the gap between traditional uses, safety, and mechanical effects.

Some questions remain, such as:

- How bioavailable are health-promoting compounds from flowers? And how do post-harvest, industrial, or culinary treatments affect such bioavailability? Can ingesting large amounts of flowers significantly raise plasma and tissue levels of health-promoting compounds?
- Can post-harvest, industrial, or culinary treatments make potentially toxic flowers edible? Can biorefinery approaches be used to obtain added-value products from potentially toxic flowers?
- What plants can be used for phytoremediation and to provide health-promoting rich flowers without posing health risks due to excessive accumulation of pollutants in edible parts?
- What amount of floral waste is necessary for its alternative circular use to be economically viable? How can smaller amounts of waste be used alternatively?
- What floral species can be used to cultivate areas with harsh edaphoclimatic conditions and/or enrich biodiversity by attracting pollinators?

## Figures and Tables

**Table 1:** Description, relevant synonymous, phytoremediation uses, toxic compounds, medicinal, and gastronomic uses of flowers.

Family [18]	Species [18]	Common Name	Place of Origin	Most Used Part/Flower Image		Phytoremediation Uses	Main Chemical Groups	Medicinal Use	Gastronomic Uses
Acanthaceae	*Aphelandra squarrosa* Nees	Aphelandra, Kuda Belang, zebra plant, saffron spike [19,20]	Central and South America [19,20]	Root, leaf [21]	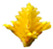	na	Alkaloids (aphelandrine, spermin), phytoanticipins (2-benzoxazolinone (BOA), 2-hydroxy-1,4-benzoxazine-3-one (HBOA)), glucosides (cyclic hydroxamic acids and their corresponding glucosides) [21,22,23,24] Note: It presents allelopathic activity [25]	Activity: antibacterial and antifungal [20,21]	Non-edible
Acanthaceae	*Justicia aurea* Schltdl.	Justicia, Yellow Jacobinia, Brazilian plume [26,27]	Central America [26]	Leaf [27,28]	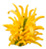	na	na	Treatment: coughs, epilepsy, anxiety, and malaria [26,27,28]	Leaf: juice [27]
Alstroemeriaceae	*Alstroemeria aurea* Graham	Amancay, Peruvian Lily, Lily of the Incas, Parrot Lily, and jingle bell [29,30]	Andean forests [30,31]	Flower, leaf, stem [29,30]	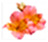	na	Glucosides (tuliposide A), tulipalin A, phenols (6-hydroxy pelargonidin glycoside)s [32]	Treatment: gynaecological and obstetric [31] Toxicity: All parts can cause skin allergies [29,32]	Non-edible [29]
Amaranthaceae	*Celosia argentea* L.	Lion hand, velvet, cockscomb, plumón, pluma, plumero rosa, cresta de gallo, celosia [33,34,35]	Asia [34], unknown origin [35]	Flower, seed, leaf [36,37]	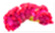	Soil decontamination [38]	Alkaloids, saponins, tannins, phenols (anthocyanin), glycoproteins [33,35,36,37,39]	Treatment: stop bleeding, liver heat, diseases of the blood, therapeutic eye diseases, and infections of the urinary tract [33,35,37]. Activity: antitumor, antiviral, hepatoprotective, immune-modulatory, antidiarrheal, anti-diabetic, anti-infective, anti-helminthic, anti-inflammatory, antioxidant, antinociceptive [33,35,36,37]	Leaf: vegetables [36] Flower: vegetables, additive food [36,39] Seed: flour [33]
Amaryllidaceae	*Allium schoenoprasum* L.	Wild chives, scallions, garlic chives, brown garlic, leaf onions, kucai [40]	Central Asia [41]	Leaf, root, flower [40,42]	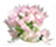	Soil decontamination (Pb, Cd, Zn, and polycyclic aromatic hydrocarbons) [43]	Phenols, terpenes (volatile and essential oils), sulphur-compounds [41,42,44,45] Low toxicity (Daily doses: 60 g FW and 120 mg essential oil [41]	Treatment: stop bleeding, lower blood pressure, and prevent infections of the urinary tract [40,44] Activity: antithrombotic, antitumor, hepatoprotective, immune-modulatory, antidiarrheal, anti-diabetic, anti-infective, antioxidant, anti-inflammatory, antimicrobial (antifungal, antibacterial, antiviral, antiprotozoal, anthelmintic) [40,41,44]	Leaf: vegetables, condiment [41,44]. All parts are edible [40]
Amaryllidaceae	*Agapanthus africanus* (L.) Hoffmanns	African Lily, Nile Lily, African agapanthus, love flower [46]	South Africa [46]	Leaf, root, flower [47]	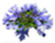	Water decontamination (TSS, COD, BOD, TP) [48]	Alkaloids (galantamine, tazatine), terpene (essential oil), tannins, phenolics (flavonoid)s, lipids (lecithin), proteins (polypeptides), saponins [46,49,50]	Treatment: heart diseases, hypertension, pregnancy and labour, cancer, and haemorrhoids [47,49,50,51]	Whole plant: infusion [50,51]
Amaryllidaceae	*Clivia miniata* (Lindl.) Bosse *Clivia miniata* var. citrina S. Watson	Bush lily, orange lily, umayime [52,53]	South Africa [54].	Whole plant [52,53,55]	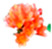	Soil decontamination (Pb and carbon).	Alkaloids (galantamine), esters (3a-4-dihydro-lactone), benzopyran ((3,4-g) indole ring system), triazines (atrazine) [52,54,55,56]	Treatment: fever, relieve pain, facilitate childbirth, and as a snake bite remedy [52,54] Activity: antimicrobial, antiviral, uterotonic, antitumor, cytotoxic activities [52,54,56] Toxicity: All plants present high toxicity (alkaloids) [55].	Non-edible [56]
Apiaceae	*Coriandrum sativum* L.	Cilantro, Chinese parsley, European coriander, cilantrillo [57]	Mediterranean regions [57]	Whole plant [58,59]	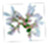	Soil decontamination (Pb, Cr and As). Water decontamination (Zn (II) ions from aqueous medium) [60,61]	Sugars, alkaloids, phenolics, resins, tannins, anthraquinones, sterols, and terpenes (essential oils) [42,57,58,59]	Activity: antimicrobial, antioxidant, anti-diabetic, anxiolytic, cardioprotective, antiepileptic, anthelmintic, antiulcer, anti-carcinogenic, diuretic, antidepressant, antimutagenic, anti-inflammatory, antilipidemic, antihypertensive, neuroprotective, diuretic [57,58,59,62] This presents cytoprotective effects in gastric epithelial cells. LD50 oil = 4.1 g/Kg [57]	Arial part: several culinary uses [57,58]
Apocynaceae	*Catharanthus roseus* (L.) G. Don	Cape vinca, chavelita, teresita, vinca rosea, Isabelita, nayon-tara [27,63]	Madagascar [53]	Leaf, root [27]	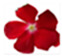	Soil decontamination (Cr, Pb, Ni and oil-contaminated soil) [64,65]	Hallucinogen (Ibogaine), alkaloids(ascartharathine, lochnenine, vindoline, vindolinenine, vincristine, vinblastine, reserpine, tetrahydroal-stronine, yohimbine, serpentine) [53,63,66] Note: the leaves have cytotoxicity [66]	Treatment: leukaemia, a popular remedy for diabetes, headache, wasp stings, sore throat, eye irritation, low blood pressure, insomnia, Hodgkin’s disease, hypertension, neuroblastoma, malaria, rhabdomyosarcoma, Wilms tumour, vascular dementia, Alzheimer’s, dermatitis, acne [27,53,63,67] Activity: antifungal [68]	Leaf: juice [27]
Apocynaceae	*Nerium oleander* L.	Adelfa, flower laurel, laurel rose, trinitaria [69]	Mediterranean regions [69]	Leaf, flower, root, stem [69]	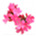	Soil decontamination (Ni and Cr) [70]	Alkaloids, tannins, steroids, terpenoids, flavonoids, saponins, and cardiac glycosides (nerifolin, peruvosid, vetoxin, thevethin A, thevethin B, ruvosid oleandrin, folinerin, adynerin, and digitoxigenin) [69,71,72] Note: Hazardous compounds (cardioactive steroids or cardiac glycosides) [55]	Treatment: cardiac affections, diabetes, rheumatic pain, epilepsy, asthma, leprosy, nervous regulation, painful menstrual periods, malaria, indigestion, ringworm, venous diseases, skin problems, warts, and chemotherapeutic agents [69,71,73] Activity: antifungal, cytotoxic, anti-inflammatory, antioxidant, analgesic, cardioprotective, neuroprotective, hepatoprotective [73] Toxicity: All plant causes abortions and skin irritant [29,55]	Non-edible [29]
Apocynaceae	*Trachelospermum jasminoides* (Lind.) Len.	Star jazmín, fake jasmíne, milk jasmine, Chinese jasmine	Asia	Leaf, flower, stem [74,75]	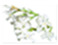	na	Lignans, alkaloids, triterpenoids, and phenolics [75,76]	Treatment: relieving rheumatic, arthritic pain, fever, gonarthritis, backache, and pharyngitis [76]. Activity: anti-inflammatory, analgesic, antitumor, antioxidant, and antimicrobial [76]	Arial part: infusion [76]
Araceae	*Aglaonema commutatum* Schott	Aglaonema, cafeto ornamental [34]	Southeast Asia [34]	Leaf, fruit [77]	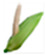	Used as a vertical greenery system (VGS) to contribute to improving air quality [78]	Alkaloids (calcium oxalate crystals, polyhydroxy alkaloids), proteins (latex), terpenes (carotenoids) [77]	Treatment: Buruli ulcer (chronic and debilitating infection of the skin) and reduced swellings [79]	Non-edible. Toxic if consumed [34]. Leaf: infusion [79]
Araceae	*Anthurium andraeanum* Linden ex André	Anthurium, capotillo, flower of love, flamenco flower	America [80]	Leaf, flower	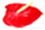	Water decontamination (COD, P, coliforms) [48]	Alkaloids (calcium oxalate crystals), glycosides (cyanogenic glycosides), and phenolics [80,81]	na	na
Araceae	*Spathiphyllum montanum* (R. A. Baker) Grayum	Spath, peace liliescuina de moisés, guisnay [20,82,83]	Tropical America [20,82,83]	Leaf [84]	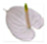	Air decontamination with toxins [83]	Phenols (flavonoids) [84]	Activity: antioxidant, anti-inflammatory, antimicrobial, anti-carcinogenic [20,84]	na
Asparagaceae	*Chlorophytum comosum* (Thunb.) Jacques	Tape, malamadre, clorofito, lasso of love, spider plant, ribbon plant [85]	Africa [86]	Leaf, flower, stem [85]	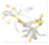	Soil decontamination (Al, Pb, Cd salt, trichloroethylene, toluene, formaldehyde, particulate matter, and benzene) [86,87,88] Air decontamination (PM) [89]	Saponin (gitogenin, ecogenin, tigogenin), glycosides and alkaloids [85,86]	Treatment: bronchitis, cough, fracture, and burns [85] Activity: antimicrobial, anti-carcinogenic, hepatoprotective, antitumour properties, and cytotoxicity against cancerous cell lines [85,86]	Arial part: infusion [90]
Asteraceae	*Bidens andicola* Kunth	Ñachac, mìshico, quello-ttica, quico, chiri chiri, zumila [20,91,92,93]	South America [20,94]	Whole plant [93,94]	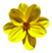	na	Alkaloids, phenolics (flavonoids), saponins, tannins, cardiotonics, steroids, terpenoids (sesquiterpene lactones), and chalcones (chalcone ester glycosides) [91,94,95,96]	Treatment: excessive vaginal fluid, postpartum, diarrhoea, cholera, stomachache, nervous afflictions, skin problems, asthma, eye inflammation, and renal affections [92,94,96] Activity: uterine antihaemorrhagic, antirheumatic, anti-inflammatory, anti-allergenic, antibacterial, antidiabetic, antimalarial, antiviral, antihypertensive, antioxidant, antimicrobial activity, and antispasmodic properties [20,94] Toxicity: It presents a moderate toxic effect [91]	Leaf: salad Whole plant: infusion [93]
Asteraceae	*Calendula officinalis* L.	Calendula, African marigold, Common marigold, Zergul, Garden Marigold, Marigold, Pot Marigold [97,98]	Southern Europe [99]	Flower [99]	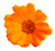	Soil decontamination (Cd, Pb) [100]	Saponins, sterols, terpenes (carotenoids, volatiles oils), tannins, resins, triterpenoids, phenols, coumarins, and quinones [42,72,97,101,102]	Treatment: used as emollient, vulnerary, moisturising, analgesic, cramps, ulcers, jaundice, and haemorrhoids [99,101] Activity: antioxidant, anti-inflammatory, antibacterial, antifungal, antiviral, antipyretic, antiseptic, antispasmodic, astringent, bitter, candidacies, cardiotonic, carminative, cholagogue, dermagenic, diaphoretic, diuretic, haemostatic, immunostimulant, lymphatic, uterotonic, and as a vasodilator [97,98,99,102,103] Toxicity: The leaves can cause phytodermatitis and cytotoxicity activity [55]	It has a slightly bitter and spicy flavour. Flower: infusion [99]
Asteraceae	*Centaurea seridis* L.	Bracera marine, thorny broom	Mediterranean region [104]	na	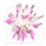	na	Glucosides, sesquiterpenoids [104,105]	Activity: anti-diabetic [104]	na
Asteraceae	*Cichorium intybus* L.	Brussels chicory, coffee chicory, root chicory, cikoria, nigana, cicoria, juju, radicheta [106,107,108]	Western Asia, Europe, and North Africa [106,107]	Whole plant [107]	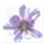	Soil decontamination with DDT (Dichlorodiphenyltrichloroethane) [88]	Sesquiterpenes lactones (lactucin, lactopicrin), aesculetin, Cichorium), coumarin (scopoletin, 6-7-dihydro coumarin, umbelliferone glycosides, terpenes (oils essential), phenolics [106,107,109]	Treatment: cardiovascular, digestive, and skin protection [107] Activity: antioxidant, hypolipidemic, anti-carcinogenic, anti-allergenic, anti-testicular, antidiabetic, diuretic, anti-inflammatory, analgesic, sedative, immunological, antimicrobial, antiprotozoal, hepatoprotective, neuroprotective, and gastroprotective [106,107]	Leaf: salad, infusion Roots: flour [107]
Asteraceae	*Chrysanthemum morifolium* Ramat	Chrysanthema	Asia [3]	Flower [3]	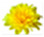	Soil decontamination with Pb	Pyrethroids (pyrethrins, deltamethrin), terpenes (sesquiterpene lactones), and phenolics (chrysanthemin) [3,110]	Treatment: used in the detoxification of blood, regulation of pressure, calming nerves, hypertension, angina, digestive system, muscular-skeletal system, respiratory system, arteriosclerosis, hypertension [3,111,112] Activity: antioxidant, anti-inflammatory, anti-carcinogenic [111] Toxicity: the flowers present phytodermatitis [55]	Flower: infusion, food supplement [3,111,113]
Asteraceae	*Coreopsis grandiflora* Hogg ex Sweet	Coreopsis	America [114]	Flower [115]	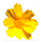	Soil disturbance [116]	Phenolics [117]	Activity: antioxidant, anti-inflammatory, antimicrobial, antimalarial, antileishmanial, and anti-Alzheimer [117]	Flower: food additive
Asteraceae	*Cota tinctoria* (L.) J. Gay	Golden marguerite, yellow chamomile [118]	Mediterranean region	Whole plant [118]	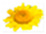	Soil decontamination with B [119]	Terpenes (volatile oils), triterpenes, tannins, and phenolics [118,120,121]	Treatment: gastrointestinal disorders, stomach, haemorrhoids, antispasmodics, stimulating menstrual flow, hepatic insufficiency, and jaundice [118] Activity: antimicrobial, anti-inflammatory, antibacterial, antispasmodic, and sedative [118,120]	Flower: meat and dairy colouring [118]
Asteraceae	*Dahlia coccinea* Cav.	Dahlia, mirasol, mountain dahlia, wild dahlia, sunflower [122]	Mexico [122,123]	Flower, root [122]	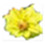	Soil decontamination with oil [64]	Terpenes (essential oils), polysaccharides (inulin), and acetylene compounds [122]	Activity: antioxidant, anti-inflammatory, anti-carcinogenic, anti-obesity, and gastroprotective [122,124]	Flower: salad, dessert, garnish Root: soup [122]
Asteraceae	*Dahlia pinnata* Cav.	Dahlia, heron flower [20]	Mexico It was declared the national flower of Mexico [20,125]	Flower, root [122]	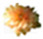	Soil decontamination oil [64]	Terpenes (essential oils), proteins (insulin), monosaccharides (fructose), acids (phytin, polyacetylenes, benzoic acid) [126] Note: Root exudates are nematode toxic	Activity: antimicrobial [20]	Flower: salad dessert, garnish Root: soup [122]
Asteraceae	*Gaillardia × grandiflora* Hort. Ex Van Houtte	Gallant, flower blanket, gold button, bloodsucker, topasa dre	na	Flower	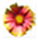	na	Alkaloids (oxalates) Note: It presents an inhibitory effect on the pathogenic fungi [127]	na	Non-edible [127]
Asteraceae	*Tagetes erecta* L.	Carnation of the moor, flower of the dead, carnation Chinese, damask, flower crest, French marigold [125,126]	Mexico The traditional day of the Dead flower in Mexico [127]	Flower [127]	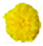	Water decontamination (textile dye blue 160), HgCl, SnCl_2_ [128]. Soil decontamination with Cd (hyperaccumulator) and oil [64,129]	Organic acids, terpenes (essential oil), alkaloids, and phenolics [125,130,131] Note: Essential oil is cytogenotoxic. It can be harmful in large amounts [132]	Treatment: therapies and aromatherapies, digestive ailments (colic, parasites, discomfort, and diarrhoea), liver diseases, antiseptic, diuretic, depurative [125,127,133] Activity: antioxidant, anti-carcinogenic, anti-inflammatory, disinfectant, healing, and antifungal [125,127,131] Toxicity: the leaves present phytodermatitis [55,127]	Flower: infusion, salad, fried. It is a natural colouring and has a bitter taste [12,113]
Asteraceae	*Taraxacum campylodes* G. E. Haglund	Dandelion, bitter chicory, diente de león [31,91,92]	Europe and Asia [120]	Whole plant [113,134]	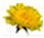	Soil decontamination (Cu, Zn, Mn, Ni, Cr, Fe, and Pb) [135,136]	Alkaloids (phytosterol, taraxacin, oxalates), phenolic (taraxastero, stigmasterol, chicoric acid, caffeic acid, acopoletin) [137,138,139,140]	Treatment: depuratives help the liver, kidney, stomachache, gall bladder, diuretic effect, constipation, clean skin impurities, acne, and hives [92,134,137,139] Activity: hepatoprotective, antirheumatic, spasmolytic, diuretic, anti-inflammatory, anti-carcinogenic, antirheumatic, anti-allergenic, anticoagulant, and anti-carcinogenic [31,137]. Toxicity: the leaves present phytodermatitis [55]	Leaf: salad, cooked Root: coffee Flower: with olive oil, cakes, fries, and wine Whole plant: infusion [113,137]
Asteraceae	*Zinnia elegans* L.	Guadalajara, mystical rose, paper flower, field chinita	Mexico and Central America [141]	Leaf, flower	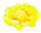	Soil decontamination (Pb and Cr) [65]	Phenols (flavonoids), glycosides, tannins, and saponins [141]	Treatment: malaria and stomach pain Activity: hepatoprotective, antiparasitic, antifungal, antibacterial, and antioxidant [141]	Flower: salad, infusion
Balsaminaceae	*Impatiens walleriana* Hook. f.	House joy, bear ears, balsam, miramelindo	Africa and Asia	Leaf, stem, flower	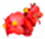	Soil decontamination with Cd (hyperaccumulator) [129]	Naphthoquinones, phenols (flavonoids), saponins (triterpenoid saponins), alkaloids (phytosterols, proteins, and terpenes (essential oils) [129,142]	Treatment: abdominal pain, ulcers, amenorrhea [129]	Flower: infusion, salad, garrison It has a sweet flavour.
Begoniaceae	*Begonia cucullata* Willd.	Begonia, sugar flower [126]	Brazil [126]	Flower [143]	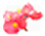	Soil decontamination with oil [64]	Phenolics, terpenes [144]	Activity: antispasmodic, astringent, ophthalmic, poultice, and stomachic activity [143]	na
Begoniaceae	*Begonia × tuberhybrida* Voss	Begonia [12,126]	Andes [126]	Flower [12]	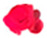	na	Alkaloids (oxalic acid, tetracyclic triterpene), phenolics [113,144]	Activity: antispasmodic, astringent, ophthalmic, poultice, and stomachic [12,143]	Petals are edible. This flower has a lemon flavour [12,113,143]
Bignoniaceae	*Tecoma capensis* (Thunb.) Lindl.	Cape Honeysuckle, tecoma [145,146]	South Africa [147]	Leaf, flower, root [148]	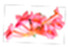	na	na It is an invasive species [147]	Treatment: pneumonia, enteritis, diarrhoea, fragrance, tonic, eliminating placenta retained in childbirth, snakebite, sleeplessness, induced sleep [148,149,150] Activity: antimicrobial, antifungal, antipyretic, antioxidant [149]	Arial part: infusion [148]
Bignoniaceae	*Tecoma stans* (L.) Juss. ex Kunth	Yellow bell, tronadora, huiztontli, huiztonxochitl [151]	Mexico [147,151]	Leaf, flower [152]	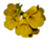	Soil and water decontamination (FeCl_3_, CaCO_3_) [128]	Alkaloids (tecomine, tecostamine), phenolics, steroids, and tannins [151,153]	Treatment: arterial hypotension, hypoglycaemia, and urinary disorder [151,152] Activity: antidiabetic, antimicrobial, and antioxidant [149,152]	Non-edible
Boraginaceae	*Heliotropium arborescens* L.	Vanilla of garden, heliotrope, grass of the mule, violoncello, cherry pie, heliotrope [154,155]	Peru	Leaf, stem, flower [155]	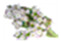	na	Esters (heliotropin, benzyl acetate), alkaloids (heliotrine, oxalates), phenols (vanillin, cynoglossin, caffeic acid), benzaldehydes (benzaldehyde, p-anisaldehyde), lithospermic acid [139,154,155]	Treatment: headache, sun stoke, sinus cancer, mucus relief, diuretic, uterine displacement, fever, migraine, high blood pressure, diarrhoea, breast cancer, kidney infection, pressure in the stomach and sternum, uterine displacement, and dysmenorrhea [139,155]	Arial part: infusion
Brassicaceae	*Alyssum montanum* L.	Spanish, garlic herb, rabies herb, rage herb	Europe [156]	Flower	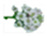	Soil decontamination (Cd, Ni, Pb, and Cu) [157,158]	Glucosinolates (goitrogenic glycosides)	na	Non-edible
Brassicaceae	*Diplotaxis tenuifolia* (L.) DC.	Rucola, yellow flower, rustic [159,160]	Mediterranean region [160]	Leaf	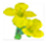	Soil decontamination with Pb [161].	Glucosinolates Note: It presents allelopathic properties (S-glucopyranosyl thiohydroximate) [162,163,164]	Treatment: digestive, diabetes, cardiovascular disorders, and cancer [159] Activity: antitumor [159]	Leaf: salad It is used in the food industry (IV gamma) [159,160]
Brassicaceae	*Matthiola incana* (L.) R. Br.	Alehí, Jasmine ashtray, White viola [134]	South Europe	Flower [134]	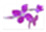	na	Isoprenoids (tocopherols), proteins (hormones), and anti-pathogens [165]	Treatment: traditional medicine, stomachache, colic, and diarrhoea for frighten [134,165]	Arial part: infusion, garnish, salad, desserts [134]
Cannabaceae	*Cannabis sativa* L.	Marijuana, marihuana, hashish, hachís, hemp [53]	Asia [53]	Whole plant [47,166]	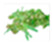	Soil decontamination (Cu, Cd, As, Ti, Cr, and Ni) [167].	Phenols (tannins), cannabinoids, terpenophenols (tetrahydrocannabinol (THC), cannabidiol (CDB)), and alkaloids [168,169]	Treatment: developmental disorder, hypertension, asthma, diabetes, heart conditions, blood pressure, epilepsy, and glaucoma [47,53,170] Activity: analgesic, antiemetic, anti-carcinogenic, antispasmodics [53] Note: the leaves are mutagenic without metabolic activation [66]	Arial part: infusion. It is a source of fibre, food, oil, and medicine [169,171]
Cannaceae	*Canna indica* L.	Achira, achira roja, achera, sago, spark, Indian cane, papantla [20,34,172]	South America [20,34,173]	Root, flowers	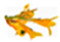	Water decontamination (Cu, Zn, fertilisers, carbamazepine, and insecticides) [48]	Alkaloids, phenols (tannins) [42,173,174]	Treatment: peptic ulcer, diarrhoea, and ulcerative colitis Activity: antibacterial, anthelmintic, antiviral, anti-inflammatory, hepatoprotective, antidiarrheal, anti-carcinogenic, analgesic, and antioxidant [20,62,173,175]	Root: starch Leaf: food cover [174]
Caryophyllaceae	*Dianthus caryophyllus* L.	Carnation, claveles [176]	Europe and Asia [176]	Flower [134]	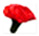	na	Triterpenes, saponins, terpenoids (carotenoids), and phenolics [42,176,177]	Treatment: HIV, simple herpes, hepatitis, vomiting, and gastric disorders [134,176,178] Activity: antibacterial, anti-fungal, antiviral, cardiotonic, diaphoretic, vermifuge, gastroprotective, anti-carcinogenic [176,179]	It has a slightly bitter flavour. Flower: salad, butter, garnish [180]
Caryophyllaceae	*Dianthus chinensis* L.	Dianthus, carnation, Chinese carnation, pae-raeng-ee-kot [181]	China [182]	Leaf, stem, flower	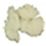	na	Phenols (eugenol), alcohols (phenyl ethyl alcohol), glycosides (melosides A and L, dianchinenosides A, B, C, and D), saponins [181,183]	Treatment: menostasis, gonorrhoea, diuretics, emmenagogue, and cough [181,182,183] Activity: anti-inflammatory, diuretic, analgesic, anti-hepatotoxic, hypotensive, anthelmintic, intestinal peristaltic, antitumor, antioxidant, antitumor, antibacterial, antifungal [181,183]	It is slightly bitter. Flower: infusion, salad, desserts, garnish [113]
Caryophyllaceae	*Gypsophila paniculata* L.	Cloud, bridal veil, paniculata, baby’s breath, sabunotu, Tibbi sabunotu [184]	Turkey, Caucasia, and Iran [185]	Leaf, stem, flower	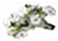	Soil decontamination (B) [185]	Allelochemical phenolic acids and saponins (triterpenoid saponins) Note: It presents insecticidal activity. It is an invasive perennial plant [184,186]	Treatment: cough, respiration system, bronchitis, stomach disorders, bone deformations, pimples, bile disorders, liver problems, rheumatism, and skin diseases [185]. Activity: antimicrobial [184]	Arial part: infusion
Caryophyllaceae	*Saponaria officinalis* L.	Soap dish, soap flower, sabunotu, tibbi sabunotu, karga sabunu, soapwort [185,187]	Turkey, Caucasia, and Iran	Root, leaf [185]	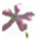	Soil and water decontamination (hydrocarbon, Cd(II), Zn(II), Cu (II) [187]	Triterpenoid, saponins (saponarioside A/B) [185,187,188]	Treatment: influenza, stomach disorders, simple herpes, bone deformations, cough, bronchitis, rheumatism, pimples, skin diseases, bile disorders and hepatic eruptions, venereal ulcers, diuretic, diaphoretic, cholagogue, and hepatic eruptions [185,187,188] Activity: anti-microbial, antipyretic, antiseptic, anthelmintic, tonic, diuretic, anti-diabetic [185,187,189]	Arial part: infusion [189]
Celastraceae	*Euonymus japonicus* Thunb.	Evonimo, bonetero	Japan [190]	Fruit, leaf, seed [190]	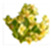	Air decontamination [191]	Alkaloids, terpenes, phenolics [190,191,192]	na	Fruit: the powder is a natural colouring for butter [190]
Convolvulaceae	*Convolvulus althaeoides* L.	Bells of the virgin, carriguela, correhuela, bindweed, leblab elhokul [193]	Mediterranean region [193,194]	Leaf, root, flowers [194,195]	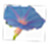	na	Alkaloids, saponins, phenolics, chlorophylls, and terpenes (carotenoids) [193] Note: Essential oil presents cytotoxic activities and is considered “weed” [194]	Treatment: wound healing, asthma Activity: laxative, purgative, antimalarial, antimicrobial, antioxidant [193,194,195]	Arial part: infusion
Convolvulaceae	*Convolvulus pseudoscammonia* C. Koch	Meadow bell, scammony Syrian bidweed, Purgin bindweed.	Asia [196]	Leaf, stem, flower [196]	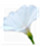	na	Alkaloids, saponins, terpenes (resin), phenols (dihydroxy cinnamic acid, flavonols), and coumarins (beta-methyl-aesculetin) [62,196,197]	Treatment: uterotonic, abortifacient, treatment of oedema, ascites, simple obesity, lung fever, ardent fever, purgative, vasorelaxant [196] Activity: antimalarial, anti-platelet aggregation, anti-carcinogenic, cell protector effect, anti-carcinogenic [194,196]	Arial part: infusion [196]
Crassulaceae	*Kalanchoe blossfeldiana* Poelln.	Kalanchoe [126]	Madagascar and East and South Africa [126,198]	Flower [198]	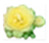	Soil decontamination with benzene [88].	Phenolics, coumarins, bufadienolides, triterpenoids, phenanthrenes, sterols, fatty acids, and kalanchosine dimalate salt [199,200] Note: All plants present high toxicity (cardioactive steroids or cardiac glycosides) [55]	Treatment: skin problems, periodontal disease, cheilitis, cracking lips in children, wounds, insect bites, ear infections, dysentery, fever, abscesses, cholera, urinary disorders, arthritis, gastric ulcers, rheumatism, pulmonary disease, rheumatoid arthritis, coughs, gastric ulcers Activity: antimicrobial [199]	Arial part: infusion
Cucurbitaceae	*Citrullus lanatus* (Thunb.) Matsum. and Nakai	Watermelon, Sandia, side, patilla [34,201,202]	Southern Africa [201,203]	Fruit, seed [53,201]	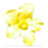	Water decontamination with Cd [204]	Saponin, alkaloids, phenols (anthocyanins, tannins, phenolic acids, flavonoids), terpenes (carotenoids, monoterpenes) [201,203]	Activity: antimicrobial, antioxidant, anti-inflammatory, antispasmodic, anti-prostatic, analgesic, antidiabetic, laxative, antiulcer, and hepatoprotective [201,202,203]	Fruit: widely used in the food industry [34]
Cucurbitaceae	*Cucurbita maxima* Duchesne	Squash, pumpkin, flor de calabacín, auyama, calabaza, sapayo, zapallo [34,92]	South Africa [34]	Fruit, leaf [202]	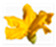	na	Saponins, alkaloids (cardenolides), and phenols (flavonoids) [202,205]	Treatment: Seed oil is used for the treatment of benign prostatic hypertrophy, laxative Activity: laxative, antimicrobial [92] Toxicity: LC50 = 4311 µg/mL	Fruit: widely used in the food industry [34]
Ericaceae	*Rhododendron simsii* Planch.	Azalea indica, azalea [206]	China [207]	Leaf, flower [207]	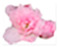	na	Alkaloids (grayanotoxane (pollen, nectar, and leaves)), phenols (flavonoids), and benzoic acid derivatives [207]	Treatment: gastrointestinal disorders, asthma, arthritis, skin diseases, cough, resolving sore toxin, amenorrhea, expectorant, and bronchitis [207] Activity: anti-inflammatory, anti-herpes, antioxidant, antiviral, hepatoprotective, and sedative [207,208]	Arial part: infusion
Euphorbiaceae	*Euphorbia milii* Des Moul.	Crown of Christ, corona de espinas, espinas de cristo [34,209]	Madagascar [34]	Whole plant	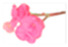	Air decontamination [210]	Terpenes (triterpenoids, diterpenoids), phenols (flavonoid, tannins s), proteins (latex) [210,211]	Treatment: respiratory tract inflammation, diarrhoea, skin ailments, gonorrhoea, tumours, cough, dysentery, asthma, hepatitis, abdominal oedema Activity: sedative and analgesic [211]	Arial part: infusion
Fabaceae	*Brownea macrophylla* Linden	Brownea, Mountain rose, Cross stick, Male cross stick [212]	Colombia, Panama, and Venezuela [213]	Flower [212]	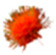	na	na	Treatment: haemostatic, for haemorrhages, birth control, and against snake bites [212]	Non-edible
Fabaceae	*Lathyrus aphaca* L.	Yellow pea, aphaca, wild pea, Indian flower [214]	Europe, Asia, Africa [214]	Flower [214]	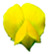	na	Seeds contain toxic amino acids [133]	na	Seed: widely used in the food industry
Fabaceae	*Senna alexandrina* Mill.	Senna, cassia, sen, cassia angustifolia [53,214]	Egypt [214,215]	Leaf, fruit, flower [53,214,216]	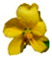	Soil decontamination (Al, Ba, Mn, and Zn)	Glycosides (anthraquinone derivatives, senna glycosides) [214,215]	Activity: laxative, antipyretic, purgative, diuretic, stomach [53,215,216]	Arial part: infusion
Fabaceae	*Senna corymbosa* (Lam.) H. S. Irwin & Barneby	Buttercup bush, Argentine Senna, sena del campo, rama negra, mata negra [20,217,218]	South America [20,217]	Flower	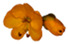	na	Glycosides (anthraquinone glycosides, naphthoquinone), phenols (flavonoids) [217]	Activity: laxative, purgative, antidiabetic, hepatoprotective, antimalarial, antipyretic, antiasthmatic, antiviral, and antibacterial [20,217,218]	Arial part: infusion
Fabaceae	*Senna didymobotrya* (Fresen.) H. S. Irwin & Barneby	Senna, popcorn cassia [219]	na	Whole plant [220,221]	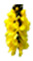	na	Alkaloids, anthraquinones, phenols (condensed tannins, hydrolysable tannins), saponins, sterols, and steroids [221]	Treatment: madness, ringworm infections, leprosy, syphilis, diabetes, convulsions, stomach complaints, wound healing, an antidote for snakebites, haemorrhoids, sickle cell anaemia. Activity: purgative, anti-inflammatory, antimalarial, hepatoprotective, antimicrobial [219,220]	Arial part: infusion
Fabaceae	*Senna papillosa* (Britton & Rose) H.S. Irwin & Barneby	Sena, candelillo	na	Flower	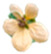	na	Phenols (flavonoids), coumarins, mellilotic acid, and iridoids	Activity: antimalarial [222]	Non-edible
Fabaceae	*Styphnolobium japonicum* (L.) Schott	Acacia from Japan, sófora, Japanase pagoda tree, Huai, Chinese scholar tree [223]	China [223]	Leaf, root, flower, seed [224]	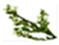	Soil decontamination (Cu, Cr, Cd, Hg, Ni, Zn and Pb) [225,226]	Alkaloids, phenols (isoflavonoids), triterpenoids [223,224]	Treatment: haemorrhoids, uterus problems, intestinal bleeding, arteriosclerosis, hypertension, cooling blood, and haemostasis [223,224] Activity: anti-inflammatory, antibacterial, antiosteoporotic, antihyperglycemic, anti-obesity, and antitumor [223,224]	Arial part: infusion [224] Flower: tea, cake [227]
Fabaceae	*Trifolium alexandrinum* L.	Clover, berseem clover [228]	Mediterranean	Whole plant [229]	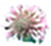	na	Cyanogenic glycosides [229]	Treatment: bronchitis, asthma, burns, cough, ulcers, sedation, polycystic ovary, heart disorders, colic Activity: anti-diabetic and laxative [229]	na
Geraniaceae	*Pelargonium domesticum* L. H. Bailey	Geranium thinking, ral geranium, malvon thinking [230]	Africa [126]	Whole plant [230]	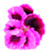	Soil decontamination with benzene [88]	na	Treatment: respiratory infection, sleep disturbance, fatigue, loss of appetite, wound healing [230] Toxicity: the leaves and stems present phytodermatitis [55]	Flower: dessert, cake, drink, salad, flower water, garnish.
Geraniaceae	*Pelargonium peltatum* (L.) L’Hér.	Gitanilla, geranium of ivy, geranio [230]	Africa [126]	Whole plant [230]	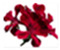	na	Phenols, terpenes (essential oil) [231]	Treatment: heal wounds Activity: antioxidant and antimicrobial [230,231,232] Toxicity: the leaves and stems present phytodermatitis [55]	Leaf has an astringent and bitter taste. The leaves and stems present gastrointestinal toxins.
Geraniaceae	*Pelargonium × hortorum* L. H. Bailey	Common geranium, malvon, garden geranium, geranio común	Africa [233]	Whole plant	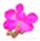	Soil decontamination (Cd and Pb) [234]	Triterpenoids, sterols, and phenols (flavonoids, anacardic acids) [235]	Treatment: respiratory infection, sleep disturbance, fatigue, loss of appetite, wound healing Activity: antioxidant and insecticidal [230,236] Toxicity: the flowers induce paralysis [237]	Flower: dessert, cake, drink, salad, flower water, garnish.
Goodeniaceae	*Scaevola aemula* R. Bronw	Fan flower	Australian [238]	Flower	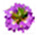	na	na	na	Arial part: infusion
Hydrangeacea	*Hydrangea petiolaris* Siebold Zuc	Hydrangea	Himalaya	Flower	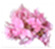	na	na	Activity: anti-inflammatory, antibacterial [239]	Arial part: infusion
Iridaceae	*Gladiolus communis* L.	Gladiolo [240]	Africa [240]	na	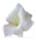	Water decontamination (Zn, Pb, Cu) [241]	na	Treatment: obesity, asthma, diabetes, and fertility	It tastes like lettuce. Flower: salad, garnish
Juglandaceae	*Pterocarya stenoptera* C. DC.	Chinese ash, Chinese wingnut, ghost maple, willow, gold trees [242,243]	China	Leaf, bark	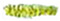	na	Phenols (tannins) [242]	Treatment: insecticide, remove scabies, eczema, abscesses, rheumatism, cold-damp bone ache, odontia, head pain, haemorrhoids, itch, pyrosis, ulcer [242,243,244] Activity: carminative, anthelmintic, anti-herpes [244]	Arial part: infusion [244]
Lamiaceae	*Agastache foeniculum* (Pursh) Kuntze	Aniseed swab, hyssop [108,245]	North America [245]	Aerial parts, seed, root [245]	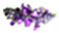	na	Note: Essential oil presents toxicity with LC50 between 18.8 to 21.6 µL/L [245,246]	Activity: antimicrobial, antiviral, antimutagenic, anti-inflammatory, and antioxidant [245]	The flower has an anise flavour.
Lamiaceae	*Lavandula angustifolia* Mill.	Alucema, lavender [126]	Mediterranean region [126,247]	Leaf, stem, flower	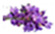	na	Terpenes (essential oils, triterpenoids, sesquiterpene, linalool, linalyl acetate), phenols (flavonoids (apigenin, luteolin)), coumarins [247,248]	Treatment: respiratory, muscular-skeletal, and cardiovascular [249] Toxicity: the leaves can produce phytodermatitis [55]	It is an aromatic herb. Arial part: infusion
Lamiaceae	*Mentha suaveolens* Ehrh.	Mastranzo, mint suaveolens [250]	Occidental Mediterranean	Leaf, stem, flower	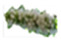	na	Terpenoids (essential oils), phenols (flavonoids) Note: It is toxic in high doses (peppermint oil) [251,252]	Activity: antimicrobial, antiviral, antioxidant, tonic, stimulating, stomachic, carminative, analgesic, choleretic, antispasmodic, sedative, hypotensive, insecticidal, analgesic, anti-inflammatory [251] Toxicity: the leaves can cause phytodermatitis [55]	It is used in the food industry. Arial part: infusion
Lamiaceae	*Mentha × piperita* L.	Peppermint, menta, hierbabuena [126]	Natural hybrid of *Mentha aquatica* and *Mentha spicata* [253]	Leaf, stem, flower	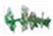		Fatty acids, terpenes, and phenolics [253]	Treatment: disorders of the mental-nervous, respiratory, digestive, metabolic, and nutritional Activity: antifungal, antiviral, antioxidant, anti-allergenic [253] Toxicity: essential oils and the leaves can cause phytodermatitis [55]	It is used in the food industry. Arial part: seasoning, cold drinks, salads [253]
Lamiaceae	*Rosmarinus officinalis* L.	Romero, Rosemary [31,108,126]	Mediterranean region [126]	Leaf, stem, flower	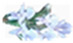	Soil decontamination (Ni, Cu, Zn, Cr, Co, Pb, and Cd) [254]	Terpenoids (essential oil: pinene, camphene, cineol, borneol, camphor) [255]	Treatment: cardiovascular, skin, muscular-skeletal diseases, sensory, nutritional, reproductive, mental-nervous, digestive, and respiratory systems [31,178,249,250,255]	Arial part: dessert, sorbet, season meet, infusion [227]
Lamiaceae	*Salvia leucantha* Cav.	Mexican bush sage or sage [256]	East Mexican and Tropical America [20]	Flower	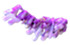	na	Diterpenoids(salvigenane and isosalvipuberulan) [257]	Treatment: mental, nervous, gastrointestinal, menstrual, digestive disorder, blood circulatory regulator [256] Activity: antibacterial, antiviral, antitumor, spasmolytic, antioxidant, anti-inflammatory [20,256]	Flower: flavouring, condiment
Lamiaceae	*Salvia microphylla* Kunth		Asia	Flower	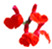	na	Terpenoids (essential oils, diterpenoids, sesquiterpenoids, triterpenoids), phenols (flavonoids) [258]	Treatment: digestive and respiratory problems	Arial part: infusion [257]
Lamiaceae	*Salvia splendens* Sellow ex Schult.	Red salvia, banderilla, salvia scarlata	Brazil [259]	Flower	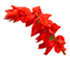	na	Terpenoids (monoterpenes, diterpenoids, sesquiterpenes, and tanshinones) and phenols (flavonoids, savianin, monardacin, and their demalonyl derivatives). Note: It presents cytotoxic activity [257,260]	Activity: antioxidant, neuroprotective, antimicrobial, antibacterial, anti-carcinogenic, anti-inflammatory, analgesic, anaesthetic, anti-stress, antiulcer, antimutagenic, antidiabetic, diuretic, haemostatic, hypoglycaemic, diaphoretic, and antidepressant [257,260,261]	It Is flavouring. Arial part: infusion, sweet-salty dishes [257]
Lamiaceae	*Vitex agnus*-castus L.	Pepper of the mountains, willow trigger	Mediterranean region	Flower	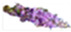	Water decontamination [262]	Phenolics, terpenes (essential oils), alkaloids [263]	Treatment: premenstrual, digestive, respiratory system, premenstrual dysphoric disorder, lactation difficulties, low fertility, menopause [249,263,264,265,266]	It is used in the food industry. Arial part: infusion [266]
Lythraceae	*Cuphea hyssopifolia* Kunth	False brecina, cufea, false Mexican heather, false erica	Central and South America [267]	Flower	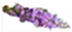	na	Phenols (tannins, flavonoids, and phenolic acids), sterols, and terpenes (triterpenes) [267] Note: It presents cytotoxic activity [268]	Treatment: stomach pain, syphilis, and cancer Activity: antiviral, antimicrobial, antioxidant, hepatoprotective, antitumor [267,268]	Arial part: infusion
Lythraceae	*Lagerstroemia indica* L.	Jupiter tree, mousse, lilac of the Indies, southern lilac, crepe	China [269]	Flower	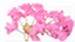	Water decontamination with fluoride [270]	Alkaloids, glycosides (anthraquinone glycosides), phenols (flavonoids), saponins [271] Note: It presents cytotoxic activity. LC50 = 60 µg/mL and LC90 = 100 60 µg/mL [272]	Treatment: stomach pain, weight loss, lower blood sugar Activity: antioxidant, antibacterial, antiviral, anti-inflammatory, anti-gout, anti-diarrheal, anti-obesity, and anti-fibrotic [271]	Arial part: infusion
Lythraceae	*Punica granatum* L.	Pomegranate, Granada, Dalim gach [27]	Asia and Mediterranean Europe [273]	Fruit, leaf [27]	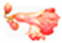	Soil decontamination [274]	Phenols (catechins), alkaloids, [273,275]	Treatment: bronchitis, tuberculosis, diarrhoea, and protecting the kidney [27,249,276] Activity: anti-carcinogenic, antimicrobial, antihypertensive, anti-diabetic, anti-HSV-1, diuretic, antioxidant [67,273,276]	Fruit: widely used in the food industry [276] Flower: infusion Leaf: fried [27]
Magnoliaceae	*Magnolia grandiflora* L.	Magnolia [126,277]	South-eastern United States [126,277]	Flower	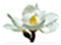	Soil decontamination (Cu, Cr, Zn, Ni, Cd, Hg) [226]	Phenols (flavonoids), terpenes (sesquiterpenes, essential oils) [277]	Treatment: flatulent dyspepsia, cough, asthma, digestive problems, and emotional distress [277] Activity: antifungal, anti-melanogenic, antioxidant and antimicrobial [277]	Flower: infusion
Malvaceae	*Ceiba speciosa* (A.St.-Hil.) Ravenna	Chorisia, Bottle tree; Drunken tree	South America	Flower	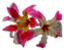	na	Phenols (flavonoids), alkaloids, coumarins, terpenes (sesquiterpenes, sesquiterpene lactones, triterpenes), steroids, lignans, cyclopropenium fatty acids, and oxidised naphthalenes [278,279]	Treatment: fever, diabetes, headache, diarrhoea, parasitic infections, rheumatism, and peptic ulcer Activity: anti-inflammatory, antimicrobial, hepatoprotective, cytotoxic, antioxidant, hypoglycaemic, and antipyretic [279]	It is used for a variety of ailments. Seed: culinary and industrial
Malvaceae	*Gossypium arboreum* L.	Cotton, cotonera, coto, algodón	Asia	Leaf, root, seed, flower	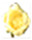	Soil decontamination with oil [64]	Phenols (gossypetin 8-o-rhamnoside, gossypetin 8-o-glucoside) and terpenes (gossypol).	Treatment: healing of wounds, ulcers, bruises, respiratory, and skin diseases	Non-edible
Malvaceae	*Hibiscus rosa-sinensis* L.	Chinese rose, cayenne, pop, hibiscus, papo, San Joaquín, carnation, Laal joba [27]	Eastern Asia National flower of Malaysia, Dominican Republic, Puerto Rico, Hawaii, Barranquilla, and Barrancabermeja (Colombia)	Flower, root, leaf [27]	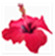	Water decontamination with zinc ions [280]	Ketones (chloroacetophenone), phenolics (tannins), steroids, proteins (mucilage) [42,72,281]	Treatment: hypertension, inflammations, dysentery, and respiratory tract [27] Activity: antispasmodic, analgesic, astringent, laxative, antioxidant, antimicrobial, anti-diabetic, cardioprotective, and anti-anxiety [281,282,283] Toxicity: the leaves can cause phytodermatitis [55]	Flower: salad, cooked, infusion Root: salad Leaf: juice [27]
Malvaceae	*Hibiscus sabdariffa* L.	Rose of Jamaica, rose of Abyssinia, hibiscus red sorrel, rosella, tart of guinea, alleluia, susur, flor de Jamaica [53,284]	West Africa [285]	Leaf, flower [285]	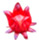	Soil decontamination (Mn and As) [286]	Phenols (protocatechuic acid) [42,284,285]	Treatment: folk remedy for abscesses, bilious conditions, cancer, cough, dysuria, cardiovascular problems, and scurvy [249,284,287] Activity: antioxidant, antiseptic, astringent, diuretic, emollient, purgative, and sedative [53,285,287]	It is a resource for food and medicine. Flower: food colouring, beverages, jams [285,287,288]
Malvaceae	*Hibiscus syriacus* L.	Altea, Syria rose, wasp, hibiscus [34]	Asia It is the national flower of Korea. Origin unknown [34,289]	Leaf, flower, root	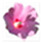	na	Terpenes (essential oils, pentacyclic triterpene esters), lignans, coumarins, and phenolics [289]	Activity: antioxidant, dermatological, anti-proliferative, anti-carcinogenic, antimicrobial, antiviral, anti-inflammatory, anti-tyrosinase [289,290]	Flower and leaf: salad, cooked, infusion
Malvaceae	*Malvaviscus arboreus* Cav	Marshmallow, false hibiscus, azocopacle, manzanita [291]	South and Central America, Southeastern United States [292]	Flower [292]	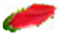	na	Phenolics, sterols, fatty acids [292]	Treatment: dysentery, stomach pain, ulcers, and coughs [292,293] Activity: antioxidant, antimicrobial, thrombolytic, anti-inflammatory, cytotoxic, hepatoprotective [292]	Arial part: infusion, salads [292]
Nyctaginaceae	*Bougainvillea spectabilis* Willd.	Bougainvillea, bogambilya, bongabilya, great bougainvillea [294]	South America [20,294]	Leaf, stem [294]	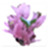	Soil decontamination (Cu, Zn) [295]	Phenols (flavonoids, tannins), saponins, sterols, triterpenes, and alkaloids [294,296]	Treatment: stomach, hepatitis, cough [294] Activity: analgesic, anti-diabetic, anti-inflammatory, antimicrobial, astringents, diuretics, antifertility [20,283,294,297]	Flower: infusion, salad, fried [294]
Nyctaginaceae	*Mirabilis jalapa* L.	Don Diego at night, dompedros, parakeet, wonder of Peru, carnation [20]	South America [20,126]	Leaf, root, flower [298,299]	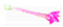	Soil decontamination (total petroleum hydrocarbons) [64]	Triterpenes, proteins, phenolics, alkaloids, and steroids [298,299]	Treatment: anthrax Activity: antitumor, virus inhibitor, anti-inflammatory, antimicrobial, antioxidant, and antidiarrheal [20,299,300]	Flower: food colouring Leaf: cooked, infusion Root: infusion Seed: infusion
Oleaceae	*Jasminum sambac* (L.) Aiton	Diamela, Arabian Jasmine, Jasmine diamela, Jasmine paper, mostia, Lily jasmine [301]	India National flower of the Philippines. It is one of the three important flowers in Indonesia [301]	Leaf, flower, root [227,301]	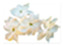	Soil decontamination with Pb [302]	Alkaloids, phenols (flavonoids, tannins), terpenoids (essential oils), coumarins, glycosides (cardiac glycosides), steroids, saponins, and phytosterols [303]	Treatment: cough, reducing sputum, cancer, uterine bleeding, ulceration, leprosy, skin diseases, and wound healing [227,301] Activity: antioxidant, anti-inflammatory, anti-carcinogenic, anti-obesity, and neuroprotective [301]	Flower: infusion, salad [227]
Onagraceae	*Fuchsia magellanica* Lam.	Fuchsia magellanica [20]	Peru, Chile, and Argentina [20]	Leaf, stem, fruit, flower	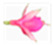	na	Phenolics [304]	Treatment: scarce menstruation and increased flow of urine [20]	It has a slightly acidic flavour. Fruit and flower: infusion
Orchidaceae	*Phalaenopsis aphrodite* Rchb. f.	Orchid	It is considered an Indonesian national flower.		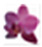	na	Alkaloids (phanaelopsin T), phenolics	Activity: antioxidant	na
Passifloraceae	*Passiflora × belotii* Pépin	Passionflower	North, Central and South America [305]	Leaf, flower, fruit	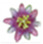	na	Phenols (flavonoids) [306]	Treatment: sedative, hypnotic, antispasmodic, and hypotensive	Flower: infusion
Plantaginaceae	*Antirrhinum majus* L.	Scrofularia, dragon mouth, bunnies, dragoncitos, gallitos [126,307]	Mediterranean region [126]	Flower	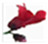	Soil decontamination (Pb and petroleum) [308,309]	Saponins, phenols [42]	Treatment: scurvy, liver disorders, tumours, haemorrhages Activity: diuretics [307]	Flower: salad [58]
Plantaginaceae	*Plantago major* L.	Llantén [38]	Europe	Leaf, seed	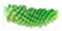	Soil decontamination (Cu, Mn, Zn, Pb and Cr) [214]	Proteins (mucilage), phenols (tannins), chromogenic glycosides (catapol), and alkaloids (noscapid) [309]	Treatment: digestive, stomach upset, intestine inflammation, abscesses, cold pimples, metabolic, muscular-skeletal, respiratory, mental-nervous, liver, kidney, rheumatism, wounds, dysentery, burns, angina, asthma, fever, tuberculosis, whooping cough, chronic renal inflammation, dermal diseases, bronchitis, purgative, arthrosis, skin problems, haemorrhoids, blood pressure, and heart afflictions [134,195,249,310,311]	Arial part: infusion [309]
Plantaginaceae	*Russelia equisetiformis* Schltdl. & Cham.	Ruselia, tears of love, firecracker, coral, fountain plant [312]	Tropical America [313]	Leaf, flower [313]	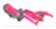	na	Sterols, triterpenes, saponins [314]	Treatment: malaria, cancer, and inflammatory diseases [313] Activity: antimicrobial [314] Toxicity: Its present cytotoxic activity [312]	Arial part: infusion [314]
Plumbaginaceae	*Limonium sinuatum* (L.) Mill.	Blue inmortelle, capitana, Straw flower, limoniun, paper flower [214]	Mediterranean region [315]	Flower [28]	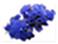	Soil decontamination with Pb [28]	na	Treatment: helps prevent the increase in glucose levels [28] Activity: antioxidant [316]	Flower: food additive, infusion [316]
Plumbaginaceae	*Plumbago auriculata* Lam.	Plumbago, Blue Jasmine, azulina, cape leadwort [34]	South Africa [34]	Flower, root [150]	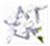	Soil decontamination (metalliferous mines and phytoremediation) [317]	Phenolics (tannins) [318]	Treatment: headache, warts, fractures, oedema, malaria, and skin lesions Activity: sedative and antimicrobial [150,319]	Arial part: infusion
Polygonaceae	*Fallopia aubertii* (L. Henry) Holub	Gabriele Falloppio, Fallopius [320]	Turkestan [320]	Flower	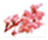	na	Phenols (flavonoids, tannins), terpenes (carotenoids, triterpenes), sterols, [320]	Activity: antioxidant, anti-carcinogenic, and antimutagenic Toxicity: It presents cytotoxic activity [320]	na
Polygonaceae	*Polygala vulgaris* L.	Common polygala	Europe	Flower	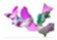	na	na	na	na
Portulacaceae	*Portulaca oleracea* L.	Verdolaga, ghotika, pinyin, krokot, little hogweed, purslane [40,126]	India	Leaf, stem, flower [40]	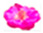	Soil decontamination with Cr (VI) [321]	Alkaloids (oxalic acid), coumarins, phenols (flavonoids, tannins), glycosides (cardiac glycosides), anthraquinones, linoleic acid, saponins [322,323]	Treatment: used in musculoskeletal, nutritional, mental-nerve, cardiovascular, haemorrhoids, and gastrointestinal disorders [40,323] Activity: anti-diarrheal, anti-inflammatory, anthelmintic, diuretic, antiasthmatic, anti-bronchitis, anti-Buruli ulcer, antioxidant, and hypoglycaemic [40,79,324]	Arial part: raw or cooked [40,323]
Ranunculaceae	*Ranunculus asiaticus* L.		Mediterranean region [325]	Flower	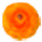	na	Alkaloids	Activity: antibacterial [326]	na
Rosaceae	*Fragaria × ananassa* (Duchesne ex Weston) Duchesne	Strawberry, fruit billa [250]	Europe	Fruit	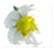	na	Phenolics, vitamin C [327]	Activity: antimicrobial, anti-allergenic, antihypertensive [327]	Fruit: widely used in the food industry
Rosaceae	*Rosa hybrid* Vill.	Rosa	na	Flower	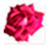	Soil decontamination (As, Co, Mo, and Ni) [328]	Phenols (glycosylated cyanidin’, pelargonidin) [329,330]	Treatment: used in respiratory and dermatological diseases and arthritis. Activity: antioxidant, anti-inflammatory laxative, and astringent [330]	It is sweet and aromatic. Flower: dessert, sweet, savoury dishes
Rubiaceae	*Gardenia jasminoides* J. Ellis	Gardenia, Cape Jasmine	Asia	Fruit [331]	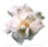	Soil decontamination (alumina and aluminium salts) [332]	Phenols (flavonoids), terpenoids, and organic acids [333]	Activity: antioxidant, anti-inflammatory, and fibrinolytic [331,333] Toxicity: the fruit can cause phytodermatitis. It presents a cytotoxic effect [55].	Fruit: food colouring [333] Flower: tea [331]
Rubiaceae	*Ixora coccinea* L.	Ixora, iosca, Santa Rita, geranium of the jungle, llama of the forests, corralito [334]	Asia [334]	Leaf, stem [334,335]	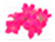	Soil decontamination [336]	Alkaloids, glycosides, phenols (flavonoids, tannins), steroids, triterpenoids, saponins, and proteins (resins) Note: It has cytotoxic activity [334]	Treatment: reduce cholesterol, control blood pressure, regeneration of tissues, reduce obesity Activity: antibacterial, antiviral, antimutagenic, anti-inflammatory, antioxidant, anthelmintic, antileishmanial, anti-asthmatic, hepatoprotective [334]	Arial part: infusion
Rubiaceae	*Palicourea marcgravii* A. St.-Hil.	Crying or golden	Brazil	Flower	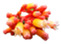	na	Alkaloids glucosides (croceaine A), triterpenes, coumarins, and phenols (phenolic acids)	Treatment: inflammation of the urinary tract Activity: antimicrobial Toxicity: It presents ictiotoxic and cytotoxic activity	Arial part: infusion
Rubiaceae	*Warszewiczia coccinea* (Vahl) Klotszch	na	Central and South America [337]	Flower	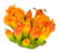	na	Triterpenes	Treatment: inhibitors of acetylcholinesterase [337]	na
Solanaceae	*Capsicum annuum* L.	Pepper, chilli, morron	Mediterranean region [338]	Fruit [79]	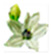	Soil and water decontamination (carbofuran residue and Pb) [339,340]	Carotenoids (capsaicin, capsorubin), alkaloids [338,341]	Treatment: Buruli ulcer and gastrointestinal benefits [79] Activity: anti-haemorrhoidal, antirheumatic, anti-inflammatory, and analgesic [341]	Fruit: macerated fruit tea, sweet and savoury dishes, salad, cooked seasoning [341]
Solanaceae	*Lycianthes rantonnetii* (Carrière) Bitter	Solano of blue flower, perennial dulcamara	Argentina and Paraguay [342]	Flower	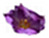	na	Alkaloids [342]	Treatment: seborrheic dermatitis, bronchitis, cough Activity: antioxidant and anti-hepatic Toxicity: It has high toxicity [342]	Arial part: infusion
Solanaceae	*Petunia × hybrida* Vilm.	Petunia [126]	The hybrid of *P. axillaris × P. integrifolia* [126]	Flower [214]	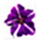	Soil decontamination (Pb, Cu, and Zn) [343]	Phenols (phenylpropanoids, anthocyanins) [344,345,346]	Activity: antimicrobial and antifungall [214,346]	Flower: garrison [214]
Solanaceae	*Solanum lycopersicum* L.	Tomato, jitomato, gold-apple [34]	Colombia, Peru, Ecuador [214]	Fruit [347,348]	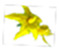	Soil decontamination (Cr, As, Zn, Cd, Pb, Cu, and Ni) [340,349]	Solanine (leaves and stems contain high concentrations) [214]	Treatment: cardiovascular diseases and macular degeneration [347,348] Activity: anti-carcinogenic, anti-furuncular [347,350] Toxicity: The leaves present phytodermatitis [55]	Fruit: widely used in the food industry [34]
Verbenaceae	*Aloysia citriodora* Palau	Kidron, lemon verbena, verbena de Indias, María Luisa, Verbena olorosa, Verbena grass Louise, Arabic tea [194,214]	America [214]	Leaf, stem, flower [92,214,351]	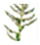	Soil decontamination (Cd and Ni) [352]	Terpenes (essential oil (neral, geranial, limonene, 1,8-cineole)), verbascosides and derivatives, and phenolics (flavonoids) Note: It has cytotoxic activity and allelopathic properties [351,353]	Treatment: digestive and nervous systems Activity: antioxidant, antifungal, antiasthmatic, antimicrobial, anaesthetic, neuroprotective, spasmolytic, anxiolytic, anti-colitis, antibacterial activity, antispasmodic, stomach, sedative, antipyretic [92,214,351,353,354]	Arial part: infusion [351] Dried leaf: marinated, seasoning, sauces [351,354]
Verbenaceae	*Lantana camara* L.	Lantana, Spanish flag, frutillo, supirrosa, cariaquito [214,355]	America [355]	Leaf, seed, flower [355]	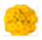	Soil decontamination (Pb, Cr, As, Zn, Cd, Cu, Hg, Ni) [356].	Alkaloids (lanthamine), terpenoids, phytosterols, saponins, phenols (tannins, phycobatannins), and steroids [42,72]	Treatment: fever, flu, stomach problems, asthma, and rheumatism [214,355,357] Activity: antispasmodic, anti-carcinogenic, antitumor, and antimicrobial [214,355,358] Toxicity: the leaves and fruits present gastrointestinal toxins [55]	Flower: infusion [355,358]
Verbenaceae	*Verbena × hybrid* Groenland & Rümpler	Verbena [143]	na	Flower [143]	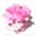	na	Phenols (flavones, flavonols)	na	Flower: raw, cooked, garnished [143]
Viola	*Viola × wittrockiana* Gams	Pansy, Wesel Ice [180]	na	Flower [180]	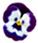	Soil decontamination (As, Cd, Pb, and Se) [307]	na	Treatment: respiratory ailments, relaxation of blood vessels, and reduction of fevers and colds [12,113] Activity: anti-inflammatory [12,359]	It has a sweet flavour [12].
Zingiberaceae	*Renealmia alpinia* (Rottb.) Maas	x’kijit, Kumpia [360,361]	Mexico [362]	Fruit [361]	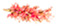	na	na	Treatment: antiemetic, antinausea, and snake venom neutraliser [360,361,363,364]	Seed: oil food [361,365]

Note: na, not available; COD, Chemical oxygen demand; PM, particulate matter; HIV, Human immunodeficiency virus; LC50, Lethal concentration; TSS, total suspended solids; BOD, Biochemical oxygen demand; TP, material contamination; PM, particulate matter; LD50, Dosage lethal media.

**Table 2:** Methods for quantifying and concentrating carotenoids and phenolics in studied plants.

Family [18]	Species [18]	Carotenoids Concentration/Quantification Technique	Phenolics Concentration/Quantification Technique
Acanthaceae	*Aphelandra squarrosa* Nees	Flower: 381.3 µg/g DW, individual carotenoids (RRLC) [366]	Root: 3.5 µmol Benzoxazinoid/g FW)/(HPLC) [22]
Acanthaceae	*Justicia aurea* Schltdl.	Flower: 47.9 µg violaxanthin/g DW (RRLC) [366]	na
Alstroemeriaceae	*Alstroemeria aurea* Graham	Flower: 4.5 to 4.9 µg total carotenoids/g DW (SM) [30]; 30 µg/g DW, individual carotenoids (RRLC) [366]; 536.6 µg/g β-carotene total carotenoids (SM) [214]	Flower: 3 mg GAE/g total phenolics (SM) [214]
Amaranthaceae	*Celosia argentea* L.	Flower: 22.1 and 116.3 µg total carotenoids/g DW, individual carotenoids (RRLC) [366] Leaf: 0.12 to 0.36 mg total carotenoids/g FW (SM) [36]	Flower: 5.01 and 6.06 g total anthocyanin/100 g FW (SM) [36], 58.4 mg GAE/g water extract, 67.6 mg GAE/g ethanol extract (SM) [39]; 13.8 and 7.7 mg total phenolics/g DW, individual phenolics (RRLC) [366] Arial part: 2.2 and 9.4 mg GAE/g DW (SM) [367], 45.2 mg GAE/g extract, and 66.7 mg QE/g extract (SM) [37]
Amaryllidaceae	*Allium schoenoprasum* L.	Flower:58.2 mg total carotenoids/kg FW (SM) [368]; 70.1 µg total carotenoids/g DW, individual carotenoids (RRLC) [366]; 423.2 µg total carotenoids/g DW [214] Root: 0.08 mg β-carotene/100 g FW, 0.65 mg total carotenoids/100 g FW (review) [40]	Flowers: 201.8 µg gallic acid/g DW, 207.3 µg coumaric acid/g DW, 887.4 µg ferulic acid/g DW, 20.3 µg rutin/g DW (HPLC) [369]; 375.8 mg total polyphenols/100 FW (SM) [368]; 9.3 mg total phenolics/g DW, individual phenolics (RRLC) [366]; 28.9 mg GAE/g DW (SM) [214] Leaf: 16.7 mg total flavonoids/g FW [42], 68.5 GAE/g (SM) [44] Root: 2.7 mg myricetin/100 g FW, 4.5 mg quercetin/100 g FW, 7.7 mg kaempferol/100 g FW, 21.0 mg GAE/100 g FW, 0.5 mg anthocyanin/100 g FW (review) [40]
Amaryllidaceae	*Agapanthus africanus* (L.) Hoffmanns	Flower: 8.1 µg total carotenoids/g DW, individual carotenoids (RRLC) [366]; 67.0 and 90.0 µg total carotenoids/g DW (SM) [214]	Flower: Identification of delphinidin, *p*-coumaroyl, kaempferol, and others (HPLC-MS) [370] 13.7 mg total phenolics/g DW, individual phenolics (RRLC) [366]; 23.6 and 25.7 mg GAE/g DW (SM) [214]
Amaryllidaceae	*Clivia miniata* (Lindl.) Bosse *Clivia miniata* var. citrina S. Watson	na	Flower: 1.8 total anthocyanin/100 mg FW (SM) [371]
Apiaceae	*Coriandrum sativum* L.	Flower: 267.6 µg total carotenoids/g DW, individual carotenoids (RRLC) [366]; 189.1 µg total carotenoids/g DW (SM) [214] Leaf: 152.8 to 169.2 mg total carotenoids/100 g DW, 38.3 to 58.6 mg β-carotene/100 g DW (before saponification), 27.1 to 47.5 mg β-carotene/100 g DW (after saponification) (SM) [372] Arial part: 2.0 g total carotenoids/kg DW, 0.6 g β-carotene/DW, 1.0 g lutein/kg DW (HPLC) [373] Seed: 1.8 to 2.2 mg total carotenoids/100 g DW, 0.3 to 0.6 mg β-carotene/100 g DW (SM) [372]	Flower: 2.4 mg total phenolics/g DW, individual phenolics (RRLC) [366]; 16.5 mg GAE/g DW (SM) [214] Seed: 12.2 GAE/g, 12.6 total flavonoids (quercetin equivalents)/g, 133.74 µg GAE/mg hydro-alcohol extract, 44.5 µg total flavonoids/mg 70% ethanol extract (SM) [42]; individual phenolics, 2.2 mg/g (HPLC-MS) [374]; individual phenolics, 129.9 mg total phenolics acids/kg DW (HPLC-MS) [58]; Arial part: individual phenolics, 6273.5 mg total phenolics/kg DW (HPLC-MS) [58]; 10.0 g total phenolics/kg DW; 0.5 g chlorogenic acid/kg DW (SM and HPLC) [373]
Apocynaceae	*Catharanthus roseus* (L.) G. Don	Flower: 3.7 µg lutein/g DW (RRLC) [366]; 163.7 and 185.1 µg total carotenoids/g DW (SM) [214] Leaf: 0.5 to 0.7 mg carotenoids/g DW (SM) [375]; 9 to 1.3 mg carotenoid/g FW, 11.9 to 32.1 mg xanthophyll/g FW (SM) [376]	Flower: 26.5 and 29.1 mg total phenolics/g DW, individual phenolics (RRLC) [366]; 67.1 and 55.5 mg GAE/g DW (SM) [214] Leaf: 05.0 to 19.0 mg phenolics/g DW (SM) [375]; 55.3 to 88.0 mg anthocyanin/g FW (SM) [376]
Apocynaceae	Nerium oleander L.	Flower: 1.0 to 3.4 µg lutein/g DW (RRLC) [366]; 51.2 to 67.6 µg total carotenoids/g DW (SM) [214] Leaf: 2.3 µmol carotenoids/g DW, 40.7 µmol β-carotene/g DW, 42.4 µmol lutein/g DW (SM) [377]	Flower: 53.8 µg GAE/mg ethanol extract, 34.3 µg quercetin/mg ethanol extract (SM) [73]; 14.0 to 22.0 mg total phenolics/g DW, individual phenolics (RRLC) [366]; 58.0 to 67.1 mg GAE/g DW (SM) [214]
Apocynaceae	*Trachelospermum jasminoides* (Lind.) Len.	Flower: 3.6 µg lutein/g DW (RRLC) [366]; 14.7 µg total carotenoids/g DW (SM) [214]	Flower: 13.2 mg taxifolin/g, 9.5 mg isoquercitrin/g, 7.6 mg chlorogenic acid/g, and 0.2 mg gallic acid/g (HPLC) [75] Aerial part: five compounds were isolated [74]; 4.1 mg total phenolics/g DW, individual phenolics (RRLC) [366]; 110.3 mg GAE/g DW (SM) [214]
Araceae	*Aglaonema commutatum* Schott	Flower: 78.7 µg total carotenoids/g DW, individual carotenoids (RRLC) [366]; 191.6 µg total carotenoids/g DW (SM) [214] Leaf: α and β-carotene [77] Fruit: lycopene, lycoxanthin, violaxanthin,α, β, γ, δ-carotene [77]; 100.0 µg β-carotene/g DW, 110.0 µg cryptoxanthin/g DW, 1100.0 µg lycopene/g DW (polarization microscopy) [378] Seed: lutein [77]	Flower: 12.5 mg GAE/g DW (SM) [367]; 0.6 mg total phenolics/g DW, individual phenolics (RRLC) [366]; 57.9 mg GAE/g DW (SM) [214]
Araceae	*Anthurium andraeanum* Linden ex André	Flower: 14.1 µg total carotenoids/g DW, individual carotenoids (RRLC) [366]; 61.9 µg total carotenoids/g DW (SM) [214]	Flower: Individual flavonoids (HPLC-MS) [80], individual flavonoids, 0.3 to 8.7 mg total anthocyanin/g FW (HPLC-ESI-MS) [81]; 12.0 to 26.0 mg flavonoids/g FW [379]; 7.7 mg total phenolics/g DW, individual phenolics (RRLC) [366]; 117.4 mg GAE/g DW (SM) [214]
Araceae	*Spathiphyllum montanum* (R. A. Baker) Grayum	Flower: 326.6 µg total carotenoids/g DW (SM) [214]	Flower: Phenols and flavonoids [84]; 87.3 mg GAE/g DW (SM) [214]
Asparagaceae	*Chlorophytum comosum* (Thunb.) Jacques	Flower: 93.9 µg total carotenoids/g DW, individual carotenoids (RRLC) [366]; 245.3 µg total carotenoids/g DW (SM) [214] Leaf: 0.5 to 3.0 mg total carotenoid/g FW (SM) [380]	Flower: 21.1 mg total phenolics/g DW, individual phenolics (RRLC) [366]; 42.7 mg GAE/g DW (SM) [214] Arial part: 1.4 mg total phenolic/g [90]
Asteraceae	*Bidens andicola* Kunth	na	Arial part: Phytochemical screening (SM) [96]
Asteraceae	*Calendula officinalis* L.	Flower: 48.0 to 276 mg carotenoids/100 g FW, 270.0 to 3510.0 mg carotenoids/100 g DW [99]; 57.2 µg total carotenoids/g FW (SM) [296]; 50.0 to 350.0 µg carotenoid/g DW [100]; 1.0 to 1.3 mg β-carotene/g DW [102]	Flower: 313.4 mg total polyphenol/g 2% flowers extract, 19.4 mg flavonoid and quercetin/g 2% flowers extract, 28.6 mg total polyphenols/g, 18.8 mg total flavonoids/g, 12.2 mg rutin and narcissin/g [42]; 0.7 to 5.1 mg GAE/g FW, 0.7 to 3.30 mg total flavonoids/g FW [296]; individual phenolics (HPLC-MS), 0.03 to 5.5 mg GAE/g DW [101]; 15.0 to 20.0 mg caffeic acid/g DW [100]
Asteraceae	*Centaurea seridis* L.	na	na
Asteraceae	*Cichorium intybus* L.	Flower: 8.0 to 30.2 µg lutein/g FW, 0.1 to 0.4 µg β-cryptoxanthin/g FW, 3.3 to 14.1 µg β-carotene/g FW [109]; 64.5 µg total carotenoids/g DW (SM) [214] Root: 1.1 to 2.4 mg lutein/kg, 0.2 to 0.5 mg β-carotene/kg (HPLC) [106]	Root: 12.8 to 101.5 mg quercetin/kg, 8.1 to 26.2 mg kaempferol/kg (HPLC-ESI-MS) [106] Flower: 20.0 to 130.0 mg GAE/100 g FW (SM) [109], 10.6 mg GAE/g FW (SM) [108]; 9.9 mg total phenolics/g DW, individual phenolics (RRLC) [366]; 46.0 mg GAE/g DW (SM) [214] Seed: 0.05 to 0.1 g total flavonoids/100 g DW; 0.5 to 2.5 g phenolic acids/100 g DW; 50.8 to 285.0 mg GAE/100 g DW; 43.3 to 150.0 mg total flavonoid/100 DW; sixty-four phenolic acids and flavonoids were extracted (SM, HPLC [42]
Asteraceae	*Chrysanthemum morifolium* Ramat	Flower: 0.1 to 0.2 mg carotenoid/g FW (SM) [381]; 11.8 to 165.5 µg lutein/g DW, 0.1 to 4.4 µg zeaxanthin/g DW, 0.1 to 1.9 µg β-cryptoxanthin/g DW, 0.1 to 2.9 µg 13-cis-β-carotene/g DW, 0.0 to 3.5 µg α-carotene/g DW, 1.4 to 21.9 g trans- β-carotene/g DW, 0.3 to 5.2 µg 9-cis- β-carotene/g DW (HPLC) [110]	Flower: 12.0 mg GAE/g DW (SM) [367]; individual phenols, 3732 to 12,562 mg total phenolics/g (HPLC-ESI) [111]; 0.5 to 5.5 mg total flavonoid/g FW, 0.2 to 2.5 total phenolic/g FW (SM) [381]; individual anthocyanin, 004 to 11.3 mg anthocyanin/g DW (HPLC-ESI-MS) [381]
Asteraceae	*Coreopsis grandiflora* Hogg ex Sweet	Flower: 1060 mg β-carotene/g (SM) [115]; 1060 µg total carotenoids/g DW (SM) [214]	Flower: 6.2 mg GAE/g DW (SM) [214]
Asteraceae	*Cota tinctoria* (L.) J. Gay	Flower: 0.7 mg α-carotene/g FW, 5.1 mg β-carotene/g FW (TLC) [115]; individual carotenoids, 46.9 mg carotenoid/100 g FW, 6.3 mg carotenoids/100 g DW (HPLC) [121]	Flower: 0.9 mg gallic acid/100 g DW, 26.8 mg chlorogenic acid/100 g DW, and other [118] Stem: 3.30 mg 4droxybenzoic acid/100 g DW, 1.3 mg caffeic acid/100 g DW, and other [118] Root: 0.01 mg quercetin/100 g DW and other (HPLC) [118]
Asteraceae	*Dahlia coccinea* Cav.	Flower: 2.5 to 24.0 g carotenoid/g DW (SM) [122]	Flower: 6.4 to 13.7 µg gallic acid/g DW, 0.9 to 4.7 µg caffeic acid/g DW, 2.7 to 16.4 µg chlorogenic acid/g DW, 3.7 to 5.8 µg hydroxybenzoic acid/g DW, 7.2 to 26.3 µg quercetin/g DW (HPLC), 2.0 to 125 mg gallic acid/g DW (SM) [122]; 86.6 mg GAE/g DW (SM) [214]; 15.7 mg total phenolics/g DW, individual phenolics (RRLC) [366]
Asteraceae	*Dahlia pinnata* Cav.	Flowers: 2.5 to 24.0 g total carotenoids/g DW (SM) [122]	Flower: 6.4 to 13.7 µg gallic acid/g DW, 0.9 to 4.7 µg caffeic acid/g DW, 2.7 to 16.4 µg chlorogenic acid/g DW, 3.7 to 5.8 µg hydroxybenzoic acid/g DW, 7.2 to 26.3 µg quercetin/g DW (HPLC), 2.0 to 125.0 mg gallic acid/g DW (SM) [122]
Asteraceae	*Gaillardia × grandiflora* Hort. Ex Van Houtte	na	na
Asteraceae	*Tagetes erecta* L.	Flower: 2.0 to 52.0 µg violaxanthin/g DW, 10.0 to 305.0 µg zeaxanthin/g DW, 0.2 to 8.2 µg luteolin/g DW, 1.0 to 36.0 µg α-carotene/g DW, 2.0 to 53.0 µg β-carotene/g DW, 1.0 to 3.8 µg 13-cis- β-carotene/g DW (HPLC) [130]; 1.9 to 11.6 mg lutein ester/g DW (HPLC) [382]	Flower: total phenolics and flavonoids, 62.3 mg GAE/g, 97.0 mg rutin equivalent/g (HPLC-MS) [131]; 27.1 to 42.2 mg GAE/g, 20.1 to 41.9 mg quercetin equivalent/g (SM) [127]; 4.6 g gallic acid/kg FW (SM) [180]
Asteraceae	*Taraxacum campylodes* G. E. Haglund	Flower: 41.9 mg total carotenoids/kg [138]; 259.0 µg carotenoids/g DW [383] Leaf: 206.4 mg total carotenoid/kg [138] Stem: 20.5 mg total carotenoid/kg [138]	Flower: 441.4 µg gallic acid/g DW, 18.7 µg rutin/g DW, 274.9 µg resveratrol/g DW, 82.9 µg vanillic acid/g DW, 593.0 µg sinapic acid/g DW (HPLC) [369]; 11.1 mg quercetin/g DW [383]; 441.1 mg gallica acid/kg DW, 274.9 mg resveratrol/kg DW, 593.0 mg sinapic acid/kg DW [384]; 22.3 GAE/g DW [140]
Asteraceae	*Zinnia elegans* L.	Flower: 223.7 to 995.9 µg total carotenoids/g DW (SM) [214]	Flower: 87.5 to 985.0 µg total anthocyanin/g FW (SM) [385]; 22.7 to 25.7 mg GAE/g DW (SM) [214] Leaf: 2.6 mg total phenolics/g DW, 0.6 mg total flavonoids/g DW (SM) [141]
Balsaminaceae	*Impatiens walleriana* Hook. f.	Flower: 148.3 µg total carotenoids/g DW (SM) [214]; 2.9 µg total carotenoids/g DW, individual carotenoids (RRLC) [366]	Flower: 6.8 mg GAE/g (SM), 96.1 mg epicatechin/100 g, 183.5 mg gallic acid/100 g, 83.9 mg protocatechuic acid/100 g [235]; 4.9 g gallic acid/kg FW (SM) [180]; 111.8 mg GAE/g DW (SM) [214]; 8.4 mg total phenolics/g DW, individual phenolics (RRLC) [366] Leaf: 3.4 µg gallic acid/g DW, 71.9 µg protocatechuic acid/g DW, 12.8 µg 4-hydroxy benzoic/g DW, 8.2 µg vanillic acid/g DW, 7.6 µg cis-p-coumaric/g DW, and other (HPLC) [386] Aerial part: individual compounds (UHPLC-MS), 12.2 mg total phenolic/g DW, 2.7 mg total phenolic acids/g DW, 3.9 mg quercetin equivalent/g DW (SM) [142]
Begoniaceae	*Begonia cucullata* Willd.	Flower: 0.03 µg total carotenoids/g FW (SM) [143]	Flower: 448.8 mg total phenolics/100 g FW (SM) [143]; 1.8 to 9.8 µg quercetin/g FW [123]
Begoniaceae	*Begonia × tuberhybrida* Voss	Flower: 0.02 µg total carotenoids/g FW (SM) [143]; 187.6 µg total carotenoids/g DW (SM) [214]; 8.7 µg total carotenoids/g DW, individual carotenoids (RRLC) [366]	Flower: 100.9 mg total phenolics/100 g FW (SM) [143]; 107.2 mg GAE/g DW (SM) [214]; 8.7 mg total phenolics/g DW, individual phenolics (RRLC) [366]
Bignoniaceae	*Tecoma capensis* (Thunb.) Lindl.	Flower: 238.6 µg total carotenoids/g DW (SM) [214]; 37.6 µg total carotenoids/g DW, individual carotenoids (RRLC) [366] Leaf: 0.3 to 0.6 mg carotenoids/g FW [145]	Flower: 19.2 mg GAE/g DW (SM) [214]; 1.3 mg total phenolics/g DW, individual phenolics (RRLC) [366] Leaf: Phytochemical screening [149]
Bignoniaceae	*Tecoma stans* (L.) Juss. ex Kunth	na	Leaf: 177.0 to 216.0 mg GAE/g DW [153] Whole plant: Phytochemical screening [387]
Boraginaceae	*Heliotropium arborescens* L.	Flower: 30.7 µg total carotenoids/g DW, individual carotenoids (RRLC) [366]	Flower: 1.2 mg total phenolics/g DW, individual phenolics (RRLC) [366]
Brassicaceae	*Alyssum montanum* L.	Flower: 238.6 µg total carotenoids/g DW (SM) [214]; 81.3 µg total carotenoids/g DW, individual carotenoids (RRLC) [366] Leaf: 0.14 to 0.16 mg total carotenoids/100 g FW (SM) [388]	Flower: 45.8 mg GAE/g DW (SM) [214]; 1.7 mg total phenolics/g DW, individual phenolics (RRLC) [366]
Brassicaceae	Diplotaxis tenuifolia (L.) DC.	Flower: 257.2 µg total carotenoids/g DW (SM) [214]; 105.4 µg total carotenoids/g DW, individual carotenoids (RRLC) [366] Leaf: individual carotenoids, 3520 and 2970 µg total carotenoid/g DW (HPLC-MS) [159]; 5.3 mg lutein/100 g, 0.7 mg violaxanthin/100 g, 0.5 mg/100 g, 0.4 neoxanthin/g (HPLC) [389]	Flower: 60.6 mg GAE/g DW (SM) [214]; 7.7 mg total phenolics/g DW, individual phenolics (RRLC) [366] Leaf: individual phenolics, 68,600 and 139,000 µg phenolics/g DW (UHPLC-Orbitrap-MS) [159]; 4.7 to 19.8 g/kg DW (HPLC-MS) [163]
Brassicaceae	*Matthiola incana* (L.) R. Br.	Flower: 200 to 1500 µg carotenoid/cm^2^ (SM) [390]; carotenoids identification by TLC and HPLC [168]; 64.6 µg total carotenoids/g DW (SM) [214]; 2.5 µg total carotenoids/g DW, individual carotenoids (RRLC) [366]	Flower: 0.1 mg GAE/g (SM), 11.1 mg protocatechuic acid (HPLC) [235]; 0.3 to 1.9 mg GAE/g [165]; anthocyanin and other flavonoids identification by TLC and HPLC [168]; 27.0 mg GAE/g DW (SM) [214]; 5.7 mg total phenolics/g DW, individual phenolics (RRLC) [366]
Cannabaceae	*Cannabis sativa* L.	Flower: 2.0 to 2.6 µg carotenoids/g FW [171]; 248.8 µg total carotenoids/g DW (SM) [214]; 19.8 µg total carotenoids/g DW, individual carotenoids (RRLC) [366]	Flower: 33.8 mg GAE/g DW (SM) [214]; 2.2 mg total phenolics/g DW, individual phenolics (RRLC) [366] Seed: 0.4 to 13.9 mg GAE/100 mg [391]
Cannaceae	*Canna indica* L.	Flower: 310.0 mg total carotenoids/kg FW, 189.0 mg total xanthophylls/kg FW, 628.0 mg xanthophylls/kg DW, 1054.0 mg total carotenoids/kg DW (HPLC) [392]; 451.5 and 2453.9 µg total carotenoids/g DW (SM) [214]	Flower: 0.06 to 1.0 mg GAE/100 g, 1.8 to 19.9 mg total flavonoid/100 g [393]; 11.0 and 12.2 mg GAE/g DW (SM) [214] Seeds: 4.8 µg flavonoids/g, 13.8 µg total polyphenols/g, anthocyanin identification [42]
Caryophyllaceae	*Dianthus caryophyllus* L.	Flowers: 1.0 to 10.0 µg total carotenoids/g FW [177]; 83.1 and 75.5 µg total carotenoids/g DW (SM) [214]; 1.6 and 15.8 µg total carotenoids/g DW, individual carotenoids (RRLC) [366] Leaf: 2.0 to 120.0 µg total carotenoid/g FW, individual carotenoids (HPLC) [177]	Flowers: 0.4 mg GAE/g (SM), 52.4 mg cyanidin-3-glucoside/100 g and 150.7 mg protocatechuic acid/100 g (HPLC) [235]; 5.3 g gallic acid/kg FW (SM) [180]; 27.4 and 48.1 mg GAE/g DW (SM) [214]; 10.9 and 15.4 mg total phenolics/g DW, individual phenolics (RRLC) [366]
Caryophyllaceae	*Dianthus chinensis* L.	Flower: 261.6 µg total carotenoids/g FW (SM) [394]; 84.2 µg total carotenoids/g DW (SM) [214] Leaf: 95.0 µg total carotenoids/g DW [395]; 0.3 to 0.5 mg carotenoid/g DW [182]	Flower: 5.3 mg GAE/g (SM), 73.2 mg catechin/100 g, 110.9 mg epicatechin/100 g (HPLC) [235]; 12.3 mg GAE/g FW, 443.5 mg total anthocyanins/100 g FW [394]; 32.6 mg GAE/g DW (SM) [214]; 9.5 mg total phenolics/g DW, individual phenolics (RRLC) [366] Leaf: 19.0 mg GAE/g DW, 65.7 total flavonoids/g DW [395]
Caryophyllaceae	*Gypsophila paniculata* L.	Flower: 80.0 to 450.0 µg β-carotene/g FW, and other carotenoids [396]; 71.8 µg total carotenoids/g DW (SM) [214]; 33.8 µg total carotenoids/g DW, individual carotenoids (RRLC) [366]	Flower: 44.6 mg GAE/g DW (SM) [214]; 22.2 mg total phenolics/g DW, individual phenolics (RRLC) [366]
Caryophyllaceae	*Saponaria officinalis* L.	Flower: 84.0 µg total carotenoids/g DW (SM) [214]; 9.5 µg total carotenoids/g DW, individual carotenoids (RRLC) [366] Leaf: 61.5 µg β-carotene/L [188]	Flower: 17.6 mg GAE/g DW (SM) [214]; 10.5 mg total phenolics/g DW, individual phenolics (RRLC) [366] Arial part: 6.5 µg GAE/mg [189]
Celastraceae	*Euonymus japonicus* Thunb.	Flower: 147.8 µg total carotenoids/g DW (SM) [214]	Flower: 88.9 mg GAE/g DW (SM) [214]; 1.9 mg total phenolics/g DW, individual phenolics (RRLC) [366] Leaf: Thirty-two compounds (HPLC-ESI-MS), individual phenolics (UPLC-TOF-MS) [190]
Convolvulaceae	*Convolvulus althaeoides* L.	Flower: 76.1 µg total carotenoids/g DW (SM) [214] Leaf: 2693.9 µg total carotenoid/g, 2655.3 µg total carotenoid/g individual carotenoids (LC-PDA-APCI/MS) [193]	Flower: 38.8 mg GAE/g DW (SM) [214]; 9.5 mg total phenolics/g DW, individual phenolics (RRLC) [366] Leaf: 3904.6 µg total phenolics/g, 2948.0 µg total phenolics/g, individual phenolics (LC-PDA-ESI/MS) [193] Whole plant: 30.3 to 125.4 mg total phenolics/g, 9.2 to 123.9 mg total flavonoids/g (SM) [397]
Convolvulaceae	*Convolvulus pseudoscammonia* C. Koch	na	na
Crassulaceae	*Kalanchoe blossfeldiana* Poelln.	Flower: 487.2 and 496.9 µg total carotenoids/g DW (SM) [214]	Flower: 21.0 to 392.0 µg anthocyanidin/g FW, 1.0 to 99.0 µg quercetin/g FW, individual phenolics (HPLC) [200]; 17 and 14.7 mg GAE/g DW (SM) [214]
Cucurbitaceae	*Citrullus lanatus* (Thunb.) Matsum. & Nakai	Flower: 335.4 µg total carotenoids/g DW (SM) [214] Fruit: 0.3 µg trans-lutein/g FW, 37.2 µg trans-lycopene/g DW, 1.5 µg trans-β-carotene/g DW (HPLC) [398]	Flower: 4.7 mg GAE/g DW (SM) [214]
Cucurbitaceae	*Cucurbita maxima* Duchesne	Flower: 1075.6 µg total carotenoids/g DW (SM) [214] Fruit: 15.4 µg β-carotene/g DW, 10.7 µg lutein/g DW, 20.6 µg violaxanthin/g DW, 9.8 µg neoxanthin/g DW (HPLC-MS) [205] Peel: Phytochemical screening [202]	Flower: 865.3 mg GAE/g DW (SM) [214] Peel: Phytochemical screening [202]
Ericaceae	*Rhododendron simsii* Planch.	Flower: 26.5 µg total carotenoids/g DW (SM) [214]; 79.0 µg total carotenoids/g DW, individual carotenoids (RRLC) [366]	Flower: 0.6 mg GAE/g (SM), 128.4 mg catechin/100 g, 65.3 mg epicatechin/100 g (HPLC) [235], 249.8 mg GAE/g DW, 20.0 mg total flavonoid/g DW (SM) [206]; 93.0 mg GAE/g DW (SM) [214]; 1.1 mg total phenolics/g DW, individual phenolics (RRLC) [366]
Euphorbiaceae	*Euphorbia milii* Des Moul.	Flower: 151.2 µg total carotenoids/g DW (SM) [214]; 7.4 µg total carotenoids/g DW, individual carotenoids (RRLC) [366] Leaf: 0.2 to 2.9 mg total carotenoid/g FW (SM) [399] Whole plant: Phytochemical screening [211]	Flower: 104.8 mg GAE/g DW (SM) [214]; 7.4 mg total phenolics/g DW, individual phenolics (RRLC) [366] Leaf: Phytochemical screening, 3.6 mg GAE/g DW, 20.3 mg quercetin/g DW [209] Whole plant: Phytochemical screening [211]
Fabaceae	*Brownea macrophylla* Linden	Flower: 377.4 µg total carotenoids/g DW, individual carotenoids (RRLC) [366]	Flower: 3.5 to 579.0 mg GAE/g DW [212]
Fabaceae	*Lathyrus aphaca* L.	Flower: 580.9 µg total carotenoids/g DW (SM) [214]	Flower: 5.7 mg GAE/g DW (SM) [214]
Fabaceae	*Senna alexandrina* Mill.	Flower: 673.5 µg total carotenoids/g DW (SM) [214]	Flower: 2.0 mg GAE/g DW (SM) [214] Whole plant: 15.9 mg gallic acid/100 g DW, 363.2 mg gentilic acid/100 g DW, 187.5 mg epigallocatechin/100 g DW, 137.7 mg kaempferol/100 g DW, and other (HPLC) [215]
Fabaceae	Senna corymbosa (Lam.) H. S. Irwin & Barneby	na	na
Fabaceae	*Senna didymobotrya* (Fresen.) H. S. Irwin & Barneby	na	Whole plant: Phytochemical screening [219]
Fabaceae	*Senna papillosa* (Britton & Rose) H.S. Irwin & Barneby	Flower: 2772.2 µg total carotenoids/g DW, individual carotenoids (RRLC) [366]	na
Fabaceae	*Styphnolobium japonicum* (L.) Schott	na	Leaf: 192.4 µg GAE/mg DW, individual phenolics (HPLC-PDA-ESI-MS/MS) [223]
Fabaceae	*Trifolium alexandrinum* L.	na	na
Geraniaceae	*Pelargonium domesticum* L. H. Bailey	Flower: 45.4 and 40.0 µg total carotenoids/g DW (SM) [214]; 0.7 and 2.1 µg total carotenoids/g DW, individual carotenoids (RRLC) [366]	Flower: 236.0 and 195.2 mg GAE/g DW (SM) [214]; 32.7 and 37.9 mg total phenolics/g DW, individual phenolics (RRLC) [366]
Geraniaceae	*Pelargonium peltatum* (L.) L’Hér.	Flower: 129.4 µg total carotenoids/g DW (SM) [214]; 3.3 µg total carotenoids/g DW, individual carotenoids (RRLC) [366]	Flower: 19.4 mg GAE/g, 2.7 mg total flavonoids/g (SM), thirty-one compounds (HPLC-MS) [231]; 139.0 mg GAE/g DW (SM) [214]; 32.4 mg total phenolics/g DW, individual phenolics (RRLC) [366]
Geraniaceae	*Pelargonium × hortorum* L. H. Bailey	Flower: 86.9 to 132.4 µg total carotenoids/g DW (SM) [214]; 3.5 µg total carotenoids/g DW, individual carotenoids (RRLC) [366] Leaf: 383.4 to 528.4 µg total carotenoids/g FW (SM) [233]	Flower: 2.4 mg GAE/g (SM), 819.0 mg homogentisic acid/100 g, 693.1 mg catechin/100 g (HPLC) [235]; 131.2 to 144.5 mg GAE/g DW (SM) [214]; 39.8 to 68.9 mg total phenolics/g DW, individual phenolics (RRLC) [366]
Goodeniaceae	*Scaevola aemula* R. Bronw	Flower: 208.2 µg total carotenoids/g DW (SM) [214]; 41.6 µg total carotenoids/g DW, individual carotenoids (RRLC) [366] Leaf: 4.5 to 8.5 mg total carotenoid/g DW (SM) [238]	Flower: 60.2 mg GAE/g DW (SM) [214]; 7.3 mg total phenolics/g DW, individual phenolics (RRLC) [366]
Hydrangeacea	*Hydrangea petiolaris* Siebold Zuc	Flower: 37.5 µg total carotenoids/g DW (SM) [214]; 7.3 µg total carotenoids/g DW, individual carotenoids (RRLC) [366]	Flower: 99.5 mg GAE/g DW (SM) [214]; 61.4 mg total phenolics/g DW, individual phenolics (RRLC) [366]
Iridaceae	*Gladiolus communis* L.	Flower: 117.7 µg total carotenoids/g DW (SM) [214]; 3.1 µg total carotenoids/g DW, individual carotenoids (RRLC) [366]	Flower: 49.9 mg GAE/g DW (SM) [214]; 2.4 mg total phenolics/g DW, individual phenolics (RRLC) [366]
Juglandaceae	*Pterocarya stenoptera* C. DC.	Flower: 133.8 µg total carotenoids/g DW (SM) [214]; 32.7 µg total carotenoids/g DW, individual carotenoids (RRLC) [366]	Flower: 89.7 mg GAE/g DW (SM) [214]; 18.1 mg total phenolics/g DW, individual phenolics (RRLC) [366]
Lamiaceae	*Agastache foeniculum* (Pursh) Kuntze	Flower: 83.7 µg total carotenoids/g DW, individual carotenoids (RRLC) [366] Arial parts: β-carotene, lutein, zeaxanthin, violaxanthin, antheraxanthin (HPLC) [245]	Flower: 6.6 mg total phenolics/g DW, individual phenolics (RRLC) [366] Arial parts: 1.6 mg apigenin/g [245]; 13.3 mg GAE/g FW [108]
Lamiaceae	*Lavandula angustifolia* Mill.	Flower: 0.2 µg total carotenoids/g FW (SM) [143]; 0.25 mg total carotenoids/g DW (SM) [400]; 418.6 µg total carotenoids/g DW (SM) [214]; 132.3 µg total carotenoids/g DW, individual carotenoids (RRLC) [366] Leaf: 0.3 to 0.45 mg total carotenoids/g DW (SM) [400]	Flower: 472.6 mg total phenolics/g FW (SM) [143]; 47.3 to 92.4 mg total phenolics/100 DW, 2.5 to 5.0 mg flavones/g DW, individual phenolics (HPLC) [401]; 4.0 to 6.0 mg total phenolics/g DW [400]; 29.5 mg GAE/g DW (SM) [214] Leaf: 4.0 to 5.0 mg total phenolics/g DW [400]; 5.6 mg total phenolics/g DW, individual phenolics (RRLC) [366] Whole plant: 13.3 mg GAE/g FW [108]; 1.1 to 32.7 µg total phenolics/mg [402]
Lamiaceae	*Mentha suaveolens* Ehrh.	Flower: 584.2 µg total carotenoids/g DW (SM) [214]; 149.9 µg total carotenoids/g DW, individual carotenoids (RRLC) [366] Leaf: 6.0 mg total carotenoids/100 g (SM) [253] Stem: 7.0 to 9.0 mg total carotenoids/100 g (SM) [253]	Flower: 87.1 mg GAE/g DW (SM) [214]; 3.9 mg total phenolics/g DW, individual phenolics (RRLC) [366]
Lamiaceae	*Mentha × piperita* L.	Flower: 545.5 µg total carotenoids/g DW (SM) [214]; 148.0 µg total carotenoids/g DW, individual carotenoids (RRLC) [366] Leaf: 4.0 to 7.0 mg total carotenoids/100 g (SM) [253] Stem: 4.0 to 8.0 mg total carotenoids/100 g (SM) [253]	Flower: 109.1 mg GAE/g DW (SM) [214]; 0.5 mg total phenolics/g DW, individual phenolics (RRLC) [366] Whole plant: 19.5 mg GAE/g FW [108]
Lamiaceae	*Rosmarinus officinalis* L.	Flower: 190.7 µg total carotenoids/g DW (SM) [214]; 14.9 µg total carotenoids/g DW, individual carotenoids (RRLC) [366] Whole plant: 259.0 µg total carotenoids/g DW (SM) [383] Leaf: 30.6 and 9.0 mg total carotenoids/L (SM) [255]	Flower: 115.8 mg GAE/g DW (SM) [214]; 7.7 mg total phenolics/g DW, individual phenolics (RRLC) [366] Whole plant: 33.2 mg GAE/g FW [108]; 1.9 mg quercetin/g DW (SM) [383]
Lamiaceae	*Salvia leucantha* Cav.	na	na
Lamiaceae	*Salvia microphylla* Kunth	Whole plant: 4.3 µg total carotenoids/g FW [258]	Whole plant: 2.4 mg GAE/g FW, 0.2 mg total anthocyanins/g FW [258]
Lamiaceae	*Salvia splendens* Sellow ex Schult.	Flowers: 0.04 µg total carotenoids/g FW [143]; 471.5 µg total carotenoids/g DW (SM) [214]; 5.5 µg total carotenoids/g DW, individual carotenoids (RRLC) [366] Leaf: 0.2 and 0.3 mg total carotenoids/g FW (SM) [259]	Flower: 2.6 mg GAE/g (SM), 30.6 mg cyanidin-3-glucoside/100 g, 20.3 mg protocatechuic acid/100 g (HPLC) [235]; 216.2 mg total phenolics/g FW [143]; 67.9 mg GAE/g DW (SM) [214]; 7.2 mg total phenolics/g DW, individual phenolics (RRLC) [366] Leaf: 0.2 and 0.3 g total anthocyanins/g FW (SM) [259]
Lamiaceae	*Vitex agnus*-castus L.	Flower: 73.5 µg total carotenoids/g DW (SM) [214]; 7.5 µg total carotenoids/g DW, individual carotenoids (RRLC) [366]	Flower: 62.9 mg GAE/g DW (SM) [214]; 28.9 mg total phenolics/g DW, individual phenolics (RRLC) [366] Whole plant: 5.0 to 32.0 mg total phenolics/g DW leaf, 2.0 to 20.0 mg total phenolics/g DW seeds, 1.0 to 3.0 mg total phenolics/g DW roots, and other phenolics [263]
Lythraceae	*Cuphea hyssopifolia* Kunth	Flower: 153.1 µg total carotenoids/g DW (SM) [214]; 157.0 µg total carotenoids/g DW, individual carotenoids (RRLC) [366]	Flower: 102.3 mg GAE/g DW (SM) [214]; 11.3 mg total phenolics/g DW, individual phenolics (RRLC) [366] Arial part: individual flavonoids ((Fourier transform (FTIR)) [403]
Lythraceae	*Lagerstroemia indica* L.	Flower: 74.3 µg total carotenoids/g DW (SM) [214]; 12.9 µg total carotenoids/g DW, individual carotenoids (RRLC) [366] Leaf: 1.2 to 1.9 mg total carotenoids/g FW (SM) [269]	Flower: 108.1 mg GAE/g DW (SM) [214]; 3.1 mg total phenolics/g DW, individual phenolics (RRLC) [366] Whole plant: Phytochemical screening [272]
Lythraceae	*Punica granatum* L.	Fruit: 0.5 to 10.1 mg total carotenoids/g FW, 0.5 to 10.2 mg β-carotene/g FW (SM) [404] Flower: 1434.4 µg total carotenoids/g DW (SM) [214]; 33.9 µg total carotenoids/g DW, individual carotenoids (RRLC) [366]	Flower: 179.1 mg GAE/g DW (SM) [214]; 146.9 mg total phenolics/g DW, individual phenolics (RRLC) [366]; 336.5 mg GAE/g [405], 15.2 to 25.9 mg GAE/g DW [275] Leaf: 79.7 mg GAE/100 g DW, and others (SM) [406] Seed: 0.7 mg GAE/g (SM) [405] Fruit: 4.0 to 11.8 mg total phenolics/g DW, and others (SM) [404]
Magnoliaceae	*Magnolia grandiflora* L.	Flower: 32.6 µg total carotenoids/g DW (SM) [214]; 20.4 µg total carotenoids/g DW, individual carotenoids (RRLC) [366]	Flower: individual phenolics, 32.6 to 140.6 mg sum of phenolics/g DW (HPLC-PDA-MS/MS-ESI) [277]; 1273.9 to 4836.9 µg total flavonols/g FW, and others (HPLC-ESI-MS) [407]; 40.4 mg GAE/g DW (SM) [214]; 0.7 mg total phenolics/g DW, individual phenolics (RRLC) [366]
Malvaceae	*Ceiba speciosa* (A.St.-Hil.) Ravenna	na	Whole plant: 8.7 and 9.4 mg gallic acid/g, 43.2 and 16.4 mg chlorogenic acid/g, 41.7 and 30.3 mg caffeic acid/g, 19.8 and 3.9 mg quercetin/g, 65.4 and 46.3 mg kaempferol/g [279]
Malvaceae	*Gossypium arboreum* L.	Flower: 203.3 and 206.5 µg total carotenoids/g DW (SM) [214]; 0.7 and 5.2 µg total carotenoids/g DW, individual carotenoids (RRLC) [366]	Flower: 120.7 mg GAE/g DW (SM) [214]; 5.3 and 7.9 mg total phenolics/g DW, individual phenolics (RRLC) [366]
Malvaceae	*Hibiscus rosa-sinensis* L.	Flower: 162.0 µg total carotenoids/g FW (SM) [408]; 448.3 and 624.3 µg total carotenoids/g DW (SM) [214]	Flower: 0.17 mg flavonoids/g, 0.09 mg total phenolics/g, 4104.0 µg flavonoids/g, 7.6 µg rutin/g, 361.9 µg quercetin/g, 50.7 µg kaempferol and myricetin/g, 61.5 mg GAE/100 g methanol extract, 59.3 mg GAE/100 g ethanol extract, 53.3 mg total flavonoids/100 g methanol extract, 32.3 mg total flavonoids/100 g ethanol extract [42]; 0.1 mg GAE/g (SM), 133.8 mg catechin/100 g (HPLC) [235]; [409]; 0.5 g gallic acid/100 g (SM) [410]; 186.2 to 281.2 mg total phenolics/100 g FW [408]; 6.2 and 8.5 mg GAE/g DW (SM) [214]
Lamiacea	*Ocimum basilicum* L.	Flower: 286.2 µg total carotenoids/g DW (SM) [214]; 88.9 and 504.6 µg total carotenoids/g DW, individual carotenoids (RRLC) [366] Whole plant: 55.0 to 69.0 µg total carotenoids/g [411]; 51.6 and 68.3 µg total carotenoids/g FW (SM) [258]	Flower: 47.5 mg GAE/g DW (SM) [214]; 0.2 mg syringic acid/g DW, individual phenolics (RRLC) [366] Leaf: 25.7 mg GAE/g FW [108] Flower: 15.2 mg GAE/g FW [108] Whole plant: 7.2 and 8.1 mg GAE/g FW, 0.2 and 0.06 mg total anthocyanins/g FW (SM) [258]
Malvaceae	*Hibiscus sabdariffa* L.	Flower: 126.9 mg total carotenoids/100 mg Leaf: 559.4 mg total carotenoids/100 mg Seed: 232.9 and 500.7 mg total carotenoids/100 mg Fruit: 641.9 mg total carotenoids/100 mg Calyx: 507.6 mg total carotenoids/100 mg [412]	Flower: 16.53 mg anthocyanins/g, 7.4 mg phenols/g, 3.5 mg flavonoids/g, individual phenolics [42]; 6.8 to 91.9 mg GAE/g (SM) [413]; 21.1 mg GAE/g DW (SM) [414] Leaf: 312.6 mg total phenolics/100 mg Arial part: 0.9 to 4.9 mg GAE/g (SM) [284]; individual phenolics value [287]
Malvaceae	*Hibiscus syriacus* L.	Flower: 0.8 mg total carotenoids/kg FW (SM) [415]; 35.3 µg total carotenoids/g DW (SM) [214]; 3.8 µg total carotenoids/g DW, individual carotenoids (RRLC) [366] Leaf: 112.9 mg total carotenoids/kg FW, individual carotenoids (SM) [415]	Flower: 63.4 mg rutin equivalents/g, 172.6 mg GAE/g (SM) [416]; 23.6 mg GAE/g DW (SM) [214]; 29.9 mg total phenolics/g DW, individual phenolics (RRLC) [366]
Malvaceae	*Malvaviscus arboreus* Cav	Flower: 49.5 µg total carotenoids/g DW (SM) [214]; 147.4 µg total carotenoids/g DW, individual carotenoids (RRLC) [366]	Flower: 0.3 mg GAE/g (SM), 38.1 mg catechin/100 g, 36.8 mg epicatechin/100 g, (HPLC) [235]; 64.7 mg GAE/g DW (SM) [214]; 14.8 mg total phenolics/g DW, individual phenolics (RRLC) [366]
Nyctaginaceae	*Bougainvillea spectabilis* Willd.	Flower: 323.6 µg total carotenoids/g DW (SM) [214]; 45.5 µg total carotenoids/g DW, individual carotenoids (RRLC) [366]	Flower: 6.9 mg GAE/g (SM), 79.6 mg catechin/100 g, 89.3 mg epicatechin/100 g, 39.8 mg gallic acid/100 g (HPLC) [235]; 1.7 to 2.3 mg GAE/g FW, 0.9 to 1.3 mg total flavonoids/g FW (SM) [296]; 15.8 mg total phenolics/g DW, individual phenolics (RRLC) [366] Leaf: 3.9 mg GAE/g [294]; 24.0 mg GAE/g DW (SM) [214]
Nyctaginaceae	*Mirabilis jalapa* L.	Flower: 524.1 µg total carotenoids/g DW (SM) [214]; 4.1 µg total carotenoids/g DW, individual carotenoids (RRLC) [366]	Flower: 88.6 mg GAE/g DW (SM) [214]; 9.5 mg total phenolics/g DW, individual phenolics (RRLC) [366] Arial part: 4.4 mg total flavonoid/g DW (SM) [417] Whole plant: individual phenolics, 2977.4 µg total phenolics/mg flowers, 304.3 µg total phenolics/mg herb, 67.9 µg total phenolics/g fruits, 12.4 µg total phenolics/mg roots [298]
Oleaceae	*Jasminum sambac* (L.) Aiton	Flower: 80.3 µg total carotenoids/g DW (SM) [214]; 4.9 µg total carotenoids/g DW, individual carotenoids (RRLC) [366]	Flower: 39.3 mg GAE/g DW (SM) [214]; 9.2 mg total phenolics/g DW, individual phenolics (RRLC) [366]
Onagraceae	*Fuchsia magellanica* Lam.	Flower: 95.3 µg total carotenoids/g DW (SM) [214]; 11.8 µg total carotenoids/g DW, individual carotenoids (RRLC) [366]	Flower: individual anthocyanins [304]; 191.7 mg GAE/g DW (SM) [214]; 42.5 mg total phenolics/g DW, individual phenolics (RRLC) [366]
Orchidaceae	*Phalaenopsis aphrodite* Rchb. f.	Flower: 50.3 and 257.8 µg total carotenoids/g DW (SM) [214]; 8.3 and 44.4 µg total carotenoids/g DW, individual carotenoids (RRLC) [366]	Flower: 22.7 and 44.6 mg GAE/g DW (SM) [214]; 7.9 and 39.9 mg total phenolics/g DW, individual phenolics (RRLC) [366]
Passifloraceae	*Passiflora × belotii* Pépin	Flower: 100.3 µg total carotenoids/g DW (SM) [214]; 49.1 µg total carotenoids/g DW, individual carotenoids (RRLC) [366]	Flower: 25.2 mg GAE/g DW (SM) [214]; 6.4 mg total phenolics/g DW, individual phenolics (RRLC) [366]
Plantaginaceae	*Antirrhinum majus* L.	Flower: 71.6 µg total carotenoids/g DW (SM) [214]	Flower: 10.0 mg GAE/g DW, 1.8 mg total flavonoids/g DW [307]; 74.7 mg GAE/g DW (SM) [214]; 8.9 mg total phenolics/g DW, individual phenolics (RRLC) [366]
Plantaginaceae	*Plantago major* L.	Flower: 355.0 µg total carotenoids/g DW (SM) [214]; 168.2 µg total carotenoids/g DW, individual carotenoids (RRLC) [366]	Flower: 76.4 mg GAE/g DW (SM) [214]; 22.8 mg total phenolics/g DW, individual phenolics (RRLC) [366] Whole plant: 1.6 mg GAE/g DW [367]
Plantaginaceae	*Russelia equisetiformis* Schltdl. & Cham.	Flower: 173.1 µg total carotenoids/g DW (SM) [214]	Flower: 31.6 mg GAE/g DW (SM) [214] Leaf: 26 phenolics (LC-MS) [418]
Plumbaginaceae	*Limonium sinuatum* (L.) Mill.	Flower: 21.41 µg total carotenoids/g DW (SM) [214]; 3.2 µg total carotenoids/g DW, individual carotenoids (RRLC) [366]	Flower: 34.2 mg GAE/g DW (SM) [235]; 5.6 to 61.4 mg GAE/g DW [315]; 31.5 mg GAE/g DW (SM) [214]; 2.0 mg total phenolics/g DW, individual phenolics (RRLC) [366]
Plumbaginaceae	*Plumbago auriculata* Lam.	na	Flower: 59.8 mg total phenolics/g DW, individual phenolics (RRLC) [366] Leaf: 24.3 mg GAE/g DW; 87.1 mg total flavonoid/g DW (SM) [318]
Polygonaceae	*Fallopia aubertii* (L. Henry) Holub	Flower: 19.9 µg total carotenoids/g DW (SM) [214]; 3.7 and 5.4 µg total carotenoids/g DW, individual carotenoids (RRLC) [366] Whole plant: phytochemical screening [320]	Flower: 66.3 mg GAE/g DW (SM) [214]; 1.7 and 2.7 mg total phenolics/g DW, individual phenolics (RRLC) [366] Whole plant: phytochemical screening [320]
Polygonaceae	*Polygala vulgaris* L.	Flower: 29.5 µg total carotenoids/g DW (SM) [214]; 1.7 µg total carotenoids/g DW, individual carotenoids (RRLC) [366]	Flower: 40.4 mg GAE/g DW (SM) [214]; 4.5 mg total phenolics/g DW, individual phenolics (RRLC) [366]
Portulacaceae	*Portulaca oleracea* L.	Flower: 476.5 to 949.5 µg total carotenoids/g DW (SM) [214]; 632.9 and 1012.4 µg total carotenoids/g DW, individual carotenoids (RRLC) [366] Arial part: 0.9 mg β-carotene/100 g FW, 5.5 mg total carotenoids/100 g FW [40]; 45.0 mg total carotenoids/100 g [324]; 18.9 to 36.5 mg total carotenoids/g DW [323]	Flower: 2.0 to 88.4 mg GAE/g DW (SM) [214]; 4.3 mg total phenolics/g DW, individual phenolics (RRLC) [366] Whole plant: 14.1 mg GAE/g DW (SM) [367], 0.3 mg quercetin/100 g FW (HPLC), 82.7 mg GAE/100 g FW, 0.24 mg total anthocyanin/100 g FW (SM) [40] Arial part: 5.9 mg total phenolics/g [90]; 3.2 mg total phenolics/100 g, 6.2 mg total flavonoids/100 g, and other phenolics [324]; 56.2 to 142.2 mg GAE/g DW [323]
Ranunculaceae	*Ranunculus asiaticus* L.	Flower: lutein and zeaxanthin (HPLC) [325]	Flower: Individual flavonoids, anthocyanidin and flavonol aglucone (HPLC) [325]
Rosaceae	*Fragaria* × *ananassa* (Duchesne ex Weston) Duchesne	Flower: 7.2 µg total carotenoids/g DW (SM) [214]	Flower: Anthocyanidins and flavonols/UHPLC-qTOF-MS [419]; 143.0 mg GAE/g DW (SM) [214]; 41.6 mg total phenolics/g DW, individual phenolics (RRLC) [366]
Rosaceae	*Rosa hybrid* Vill.	Flower: 80.8 to 106.2 µg total carotenoids/g DW (SM) [214]; 33.2 and 64.2 µg total carotenoids/g DW, individual carotenoids (RRLC) [366]	Flower: 2.6 mg GAE/g (SM), 880.0 mg homogentisic acid/100 g, 587.9 mg protocatechuic acid/100 g, 825.7 mg epicatechin/100 g (HPLC) [235]; quercetin and kaempferol compounds [329]; 106.3 to 153.4 mg GAE/g DW (SM) [214]; 7.8 to 19.2 mg total phenolics/g DW, individual phenolics (RRLC) [366]
Rubiaceae	*Gardenia jasminoides* J. Ellis	Flower: 21.8 µg total carotenoids/g DW (SM) [214]; 6.9 µg total carotenoids/g DW, individual carotenoids (RRLC) [366]	Flower: 29.6 mg GAE/g DW (SM) [214]; 1.9 mg total phenolics/g DW, individual phenolics (RRLC) [366] Leaf: 9.1 mg gallic acid/100 g DW, 141.0 mg catechin/100 g DW, 72.1 mg rutin hydrate/100 g DW, 19.1 mg quercetin/100 g DW (HPLC) [331] Whole plant: 17.3 mg GAE/g (SM) [367]
Rubiaceae	*Ixora coccinea* L.	Flower: 387.9 µg total carotenoids/g DW (SM) [214]	Flower: 9.5 mg GAE/g DW (SM) [214] Whole plant: 210.6 µg GAE/mg flower, 180.6 µg GAE/mg leaf, 100.3 µg GAE/mg stem [420]
Rubiaceae	*Palicourea marcgravii* A. St.-Hil.	na	na
Rubiaceae	*Warszewiczia coccinea* (Vahl) Klotszch	Flower: 97.2 µg total carotenoids/g DW, individual carotenoids (RRLC) [366]	Whole plant: 595.0 mg GAE/100 g DW (SM) [421]
Solanaceae	*Capsicum annuum* L.	Flower: 482.6 µg total carotenoids/g DW (SM) [214]; 139.7 µg total carotenoids/g DW, individual carotenoids (RRLC) [366]	Flower: 65.6 mg GAE/g DW (SM) [214]; 9.7 mg total phenolics/g DW, individual phenolics (RRLC) [366] Fruit: 1012.0 to 4135.5 µg GAE/g FW (SM) [42]
Solanaceae	*Lycianthes rantonnetii* (Carrière) Bitter	na	na
Solanaceae	*Petunia × hybrida* Vilm.	Flower: 0.1 to 35.8 µg β-carotene/g FW, 0.0 to 13.9 µg lutein/g FW, and others (HPLC) [422]; 80.8 to 213.0 µg total carotenoids/g DW (SM) [214]; 9.1 µg total carotenoids/g DW, individual carotenoids (RRLC) [366]	Flower: 30.2 to 48.9 mg GAE/g DW (SM) [214]; 4.8 mg total phenolics/g DW, individual phenolics (RRLC) [366]
Solanaceae	*Solanum lycopersicum* L.	Flower: 748.1 µg total carotenoids/g DW (SM) [214]; 88.1 µg total carotenoids/g DW, individual carotenoids (RRLC) [366]	Flower: 47.8 mg GAE/g DW (SM) [214]; 3.4 mg total phenolics/g DW, individual phenolics (RRLC) [366]
Verbenaceae	*Aloysia citriodora* Palau	Flower: 50.4 µg total carotenoids/g DW (SM) [214]; 1.7 µg total carotenoids/g DW, individual carotenoids (RRLC) [366]	Flower: 39.1 mg GAE/g DW (SM) [214]; 15.6 mg total phenolics/g DW, individual phenolics (RRLC) [366] Leaf: 260.0 to 350 µg GAE/g DW, 12.0 to 13.0 µg total flavonoids/g DW (SM) [353]
Verbenaceae	*Lantana camara* L.	Flower: β-carotene [5]; 64.8 to 2056.1 µg total carotenoids/g DW, individual carotenoids (RRLC) [366]	Flower: 0.1 mg GAE/g (SM), 56.1 mg catechin/100 g, 48.6 mg cyanidin-3-glucoside/100 g (HPLC) [235]; 10.9 to 22.5 mg total phenolics/g DW, individual phenolics (RRLC) [366] Leaf: 11.1 mg flavonoids/g, 917.6 mg polyphenol/100 g, 328.6 mg polyphenols/100 g [42] Stem: 3.29 mg flavonoids/100 g, 8.0 mg flavonoids/100 g [42]
Verbenaceae	*Verbena* × *hybrid* Groenland & Rümpler	Flower: 0.05 µg total carotenoids/g FW (SM) [143]; 13.3 to 74.2 µg total carotenoids/g DW (SM) [214]; 12.7 to 106.3 µg total carotenoids/g DW, individual carotenoids (RRLC) [366]	Flower: 809.0 mg total phenolics/100 g FW (SM) [143]; 35.1 to 44.4 mg GAE/g DW (SM) [214]; 21.3 to 797.0 mg total phenolics/g DW, individual phenolics (RRLC) [366]
Viola	*Viola* × *wittrockiana* Gams	Flower: β-carotene, lycopene, and xanthophyll (FT-Raman) [423]	Flower: Kaempferol, quercetin dihydrate, lutein, and other anthocyanins (FT-Raman) [423]; 64.0 mg total phenolics/g extract LC-DAD-ESI/MS [424]; 15.20 mg GAE/g DW, 2.3 mg total flavonoids/g, individual phenolics DW [307]; 5.1 g GAE/kg FW [180]
Zingiberaceae	*Renealmia alpinia* (Rottb.) Maas	Flower: 3044.7 µg total carotenoids/g DW, individual carotenoids (RRLC) [366]	Fruit: 9.2 mg GAE/g DW (MS), individual anthocyanin (HPLC) [365]

Note: na, not available; DW: dry weight; FW, fresh weight; SM, Spectrophotometric method; GAE, gallic acid equivalents; QE, quercetin equivalent; RRLC, Rapid resolution liquid chromatography; HPLC, High performance liquid chromatography; MS, mass spectrometry; ESI, electrospray ionisation; TOF-MS, time-of-flight mass spectrometry; HPLC, High-performance liquid chromatography.

**Table 3:** Some common carotenoids present in flowers.

Carotenoids	Edible Flowers
Antheraxanthin	*Agapanthus africanus* [366], *Agastache foeniculum*, *Agastache rugosa* [425], *Calendula officinalis* ‘Alice Orange’ [426], *Calendula officinalis* ‘Alice Yellow’, *Camelia chrysantha* [5], *Campanula shetleri*, *Campanula carpatica*, *Capsicum annuum* [366], *Celosia cristata* [35], *Coriandrum sativum*, *Cuphea hyssopifolia*, *Diplotaxis tenuifolia*, *Eschscholzia californica*, *Fuchsia magellanica*, *Gazania* spp. ‘Daybreak Orange’, *Gazania* spp. ‘Day Break Yellow’, *Gentiana lutea* [5], *Gypsophila paniculate* [366], *Helianthus annuus* ‘Sunrich Orange’ [5], *Inula helenium* [427], *Lantana camara* [366], *Lilium* spp. ‘Connecticut King’ [426], *Lilium* spp. ‘Montreux’, *Lilium* spp. ‘Saija’, *Lilium tigrinum* ‘Red Night’, Lily Connecticut king, *Lotus japonicus* [5], *Malvaviscus arboreus* [366], *Mimulus lewisii*, *Minulus cupreus*, *Mimulus cardinalis*, *Minulus verbenacreus*, *Minulus guttatus*, *Minulus kelloggil*, *Mimulus jungermannioides* [428], *Momordica charantia*, *Narcissus poeticus* ‘Florex Gold’ [5], *Nicotiana glauca* [5], *Passiflora × belotti* [366], *Petunia × hybrida* [429], *Phalaenopsis aphrodite*, *Portulaca oleracea*, *Rosa × hybrida* [366], *Scaevola aemula*, *Solanum laxum* [366], *Tagetes erecta* orange [430], *Tagetes patula* ‘Safari Tangerine’ [5], *Tecoma capensis* [366], *Tropaeolum majus*, *Verbena × hybrid*, *Viola tricolour* [5], and *Viola witrockiana* ‘Pansy’ [307].
Astaxanthin	*Adonis aestivalis*, *Adonis annua*, *Gazania rigens*, *Gerbera jamesonii* yellow, orange and red, *Hieracium aurantiacum*, *Hieracum pilosella*, *Hypochoeris radicata*, transgenic *Lotus japonicus*, *Senecio scandens*, *Tagetes erecta* yellow and orange [430], *Tagetes patula* [130].
Capsanthin	Asiatic hybrid Lily, *Lilium* spp. ‘Saija’, *Lilium* spp. ‘Red Night’, Tiger Lily [5].
α-Carotene	*Acnutea ageratum*, *Agastache foeniculum*, *Agastache rugosa* [425], *Bidens ferulaefolia*, *Calendula officinalis,* ‘Alice Orange’ and ‘Alice Yellow’ [5], *Chrysanthemum carinatum*, *Chrysanthemum segetum*, *Coreopsis grandiflora*, *Coreopsis tinctoria*, *Coreopsis verticillate*, *Cosmidium brunette*, *Begonia argentea*, twenty-three *Dendranthema grandiflorum* [110], *Dimorphotheca aurantiaca*, *Helianthus decapitatis* var. Loddon Gold, *Helichrysum bracteatum*, *Hypericum perforatum* [5], *Hypochoeris radicata*, *Lantana camara* [5,366], *Lavandula angustifolia* [366], *Layia elegans*, *Layia platyglossa*, *Momordica charantia*, Osmanthus Chenghong Dangui, *Osmanthus fragrans* [5], *Osmanthus fragrans* ‘Yanhong Gui’ [431], *Passiflora × belotti*, *Renealmia Alpinia* [366], *Rhododendron mole* [432], *Rudbeckia speciosum*, *Santolinas teretifolia*, *Santolinas viridis*, *Spathiphyllum montanum* [366], *Senecio scandens*, *Solidago* sp., *Tagetes erecta* yellow and orange [430], *Tagete patula* [130], *Taraxacum kok-saghyz*, *Tragopogon pratensis*, *Tropaeolum majus* [5], *Ursinia calenduliflora*, *Venidium decurrens*, *Zinnia elegans* [433], *Zinnia elegans* ‘Dreamland Red’, and ‘Dreamland Yellow’ [433].
β-Cryptoxanthin	*Camelia chrysantha* [426], *Canna indica* [5], *Cosmos bipinnatus*, twenty-three *Dendranthema grandiflorum* [110], *Eschscholzia California*, *Gentiana lutea* [5], *Gerbera jamesonii* yellow, orange, and red, *Hemerocallis disticha* [5], *Hieracium aurantiacum*, *Ipomoea* sp. [5], *Ipomoea obscura*, *Ipomoea nil*, *Lantana camara* [5,366], *Melampodium divaricatum*, *Mimulus tigrinus* [5], *Momordica charantia*, *Narcissus pseudonarcissus* ‘King Alfred’, *Nicotiana glauca*, *Pyrostegia venusta*, *Sandersonia aurantiaca*, *Tabebula chrysantha* [5], *Tagetes erecta*, *Tagetes erecta* yellow and orange [430], *Tagetes patula* [130], *Tecoma capensis* [214], *Tropaeolum majus* [5], *Zinnia elegans* [214], *Zinnia elegans* ‘Dreamland Red’, and ‘Dreamland Yellow’ [433].
Β-Carotene	*Adonis aestivalis*, *Agastache foeniculum*, *Agastache rugosa* [425], *Agastache anisate* [214], *Aglaonema commutatum*, *Allium schoenoprasum*, *Alyssum montanum* [366], *Antirrhinum majus* ‘Snapdragon’ [307], *Anthurium andreanum* [214,366], *Allamanda cathartica* [5], *Begonia argentea*, *Begonia cavaleriei* [366], *Borago officinalis* [434], *Boronia megastigma*, *Brassica oleraceae*, *Brownea macrophylla* [366], nine *Calendula officinalis* [426], *Calendula officinalis* ‘Alice Orange’ [5], *Calibrachoa* ‘Million Bells Yellow’ [5], *Camelia chrysantha* [426], *Campanula carpatica* [366], *Canna indica*, *Cannabis sativa*, *Capparis spinosa*, *Capsicum annuum* [366], *Carica papaya*, *Celosia argentea*, *Celosia cristata* [366], *Centaurea cyanus*, *Chlorophytum comossum*, *Coriandrum sativum* [366], *Cosmos bipinnatus*, Multiflora chrysanthemum, *Chrysanthemum morifolium* [426], *Crocus sativus*, *Cucurbita maxima*, *Cucumis sativus* var. Anguria, *Cuphea hyssopifolia* [214,366], *Cyclamen hederifolium*, *Cyclamen mirabile*, *Cyclamen persicum*, *Dahlia pinnata* [366], twenty-three *Dendranthema grandiflorum* [110], *Dianthus caryophyllus*, *Dimorphotheca aurantiaca*, *Diplotaxis tenuifolia*, *Drymonia affinis* [366], *Ethlingera elatior*, *Eustoma grandiflorum* ‘Azuma-no-yuki’ and ‘Halley Lime’, *Gazania × hybrida* [5], *Gentiana lutea* [5], *Gerbera jamesonii* yellow and orange [5], *Guzmania hybrid*, *Gypsophila paniculate* [214], *Heliotropium peruvianum* [366], *Hemerocallis disticha*, *Hibiscus sabdariffa* [435], *Hieracium aurantiacum*, *Hieracium pilosella*, *Hypochoeris radicata*, *Hypericum perforatum*, *Ipomoea* sp., *Ipomoea obscura* [5], *Ipomoea nil*, *Inula helenium* [427], *Ipomoea obscura*, *Lagerstroemia indica* [366], *Lantana camara* [5,366], *Lavandula angustifolia* [214,366], *Lilium* spp., *Lilium* spp. ‘Conneticut King’ [426], *Lilium* spp. ‘Montreux’, *Limonium sinuatum* [366], *Lycium barbarum*, *Lycoris radiata*, *Lotus japonicus*, *Melampodium divaricatum*, *Mentha × piperita* [366], *Mentha suaveolens* [366], *Mimulus cupreus*, *Mimulus trigrinus* [5], *Momordica charantia*, *Narcissus poeticus,* ‘Scarlet Elegance’, *Narcissus pseudonarcissus,* ‘King Alfred’; *Nelumbo lutea.* ‘American Yellow’, *Nicotiana glauca* [5], *Nicotiana tabacum* [432], *Ocimun basilicum* [366], *Osmanthus*, *Osmanthus fragrans* [5], *Osmanthus fragans* ‘Yanhong Gui’ [431], *Passiflora × belotti*, *Phalaenopsis aphrodite* [214,366], *Petunia × hybrida* [5,366], *Portulaca oleracea* [366], *Pyrostegia venusta* [5], *Punica granatum* [214,366], *Renealmia alpinia* [366], *Rhododendron mole* [432], *Rosa hybrida* [214,436], *Rosa hybrida* ‘Alister Stella’, *Rose* ‘American Pillar’, *Rose canina*, *Rose* ‘Golden Wings’, *Rose moyesii*, *Rose pomífera*, *Rose rubrijoli*, *Rose rubiginosa*, *Rose* ‘Saraband’, *Russelia equisetiformis* [366], *Saccharum edule*, *Sandersonia aurantiaca*, *Senna paillosa* [366], *Senecio scandens*, *Solanum laxum* [366], white *Solanum lycopersicum*, *Solanum rantonnetii* [366], *Sophora japonica* [214], *Sphatiphyllum montanum* [366], *Tagetes erecta* yellow and orange [430], *Tagetes patula* [130], *Tobebuia chrysantha*, *Thumbergia alata* [5], and *Trifolium hybridum*, orange and red *Tropaeolum majus* [437], *Tulipa* sp. ‘Golden Harvest’, *Verbena × hybrida* [214], *Viola tricolor*, *Viola witrockiana* ‘Pansy’ [307], *Vitex agnus-castus* [366], *Zinnia elegans*, *Zinnia elegans* ‘Dreamland Red’, and ‘Dreamland Yellow’ [5,433].
Lutein	*Agapanthus africanus*, *Agastache anisate* [366], *Agastache foeniculum*, *Agastache rugosa* [425], *Aglaonema commutatum* [366], *Allamanda cathartica* [5], *Allium schoenoprasum* [366], *Aloysia citrodora*, *Alyssum montanum*, *Anthurium andraeanum* [366], *Antirrhinum majus* ‘Snapdragon’ [307], *Aphelandra squarrosa*, *Begonia argentea* [366], *Begonia × tuberhybrida*, *Boronia megastigma*, *Bidens ferulifolia*, *Bougainvillea spectabilis*, *Borago officinalis* [434], *Calendula officinalis* ‘Alice Orange’ and ‘Alice Yellow’, *Calibrachoa* ‘Million Bells Yellow’ [5], *Campanula carpatica* [366], *Campanula shertleri*, *Cannavis sativa* [366], *Capparis spinosa*, *Capsicum annuum* [366], *Capparis spinose*, *Canna indica* [5], *Cannabis sativa*, red *Catharanthus roseus*, *Celosia argentea*, *Celosia cristata* [366], *Centaurea cyanus*, *Chlorophytum comossum* [366], *Chrysanthemum* spp., Sunny Orange Chrysanthemum, yellow Paragon *Chrysanthemum morifolium* [5], *Cichorium intybus*, *Coeopsis grandiflora*, *Convolvulus scammonia*, *Coriandrum sativum* [366], *Cosmos bipinnatus*, *Cucumis sativas* var. Anguria, *Cuphea hyssopifolia* [366], *Cyclamen hederifolium*, *Cyclamen mirabile*, *Cyclamen persicum* [438], twenty-three *Dahlia pinnata* [366], *Dendranthema grandiflorum* [110], *Dianthus caryophyllus*, *Drymonia affinis* [366], *Drymonia* sp., *Escallonia rubra*, *Euonymus japonicus*, *Euphorbia milii*, *Eustoma*, *Eustoma grandiflorum*, *Eustoma grandiflorum* ‘Azuma-no-yuki’ and ‘Halley Lime’, *Fallopia aubertii*, *Fuchsia magellanica*, *Gladiolus communis*, *Gazania* spp., ‘Day Break Yellow’ [5], *Gentiana lutea* [426], *Gerbera jamesonii* ‘Dancer’ [5], *Gladiolus communis*, *Gossypium arboreum*, *Guzmania hybrid*, *Gypsophila paniculates* [366], *Helianthus annuus*, *Helianthus annuus* ‘Sunrich Orange’ [5], *Heliotropium peruvianum* [366], *Hemerocallis disticha* [5], *Hibiscus sabdariffa* [435], *Hibiscus syriacus*, *Hydrangea petiolaris*, *Impatiens balsamina*, *Impatiens walleriana*, *Inulina helenium* [427], *Ipomoea* sp., *Ipomoea nil*, *Ipomoea obscura* [5], *Ixora coccinea*, *Jasminum sambac*, *Kalanchoe blossfeldiana*, *Lagerstroemia indica* [366], *Lantana camara*, *Lavandula angustifolia* [366], *Lilium* spp. [5], Lily connecticut, *Lilium* spp. ‘Cocceticut King’ [426], *Lilium* spp. ‘Montreux [5]‘, *Lycoris radiata*, *Lotus japonicus*, *Malvaviscus arboreun*, *Mattiola incana*, *Melampodium divaricatum*, *Mentha × piperita*, *Mentha suaveolens* [366], *Mirabilis jalapa*, *Momordica charantia*, *Narcissus poeticus* ‘Scarlet Elegance’, *Narcissus pseudonarcissus* ‘King Alfred’, *Narcissus pseudonarcissus* [5], *Nerium oleander*, *Nelumbo lutea,* ‘American Yellow’, *Nicotiana glauca* [5], *Nicotiana tabacum* [432], *Ocimun basilicum* [366], *Osmanthus jingui*, *Osteospermum ecklonis* ‘Jury’, *Osteospermun fruticosum*, *Papaver rhoeas*, *Passiflora × belotti* [366], *Pelargonium × domesticum*, *Pelargonium × hortorum*, *Pelargonium peltatum*, *Petunia × hybrida* [5,366], *Phalaenopsis aphrodite* [366], *Pterocaya stenoptera*, *Petunia × hybrida* [429], *Plantago major*, *Polygala vulgaris*, *Punica grantum*, *Purtulaca oleracea* [366], *Pyrostegia venusta* [5], *Rhododendron mole* [432], *Rhododendron simsii*, *Rosa hybrida* [366], *Rose* ‘American Pillar’, *Rose* ‘Golden Wings’, *Rose moyesii*, *Rosmarinus officinalis*, *Rubiaceae warszewiczia*, *Russelia equisetiformis* [366], *Saintpaulia ionantha*, *Salvia splendens*, *Saponaria officinalis*, *Scaevola aemula*, *Senna alexandrina*, *Senna papillosa*, *Solanum laxum* [366], *Solanum lycopersicum* ‘M82’ [5], *Sophora japónica*, *Spathiphyllum montanum* [366], *Solanum lycopersicum*, *Solanum rantonnetii* [366], *Tabebuia chrysantha*, *Tagetes erecta* [5], Lady *Tagetes erecta*, *Tagetes patula* [430], *Tagetes patula* ‘Safari Tangerine’ [5], *Tecoma capensis*, *Trachelospermum jasminoides*, *Theretia peruviana*, *Thumbergia alata* [5], *Trachelospermum jasminoides*, *Trifolium cernnum*, *Trifolium hybridum*, *Tropaeolum majus* [437], *Tulipa* sp. ‘Golden Harvest’ [5], *Verbena × hybrida* [366], *Viola tricolor*, *Viola witrockiana* ‘Pansy’ [307], *Vitex agnus-costus* [366], *Warszewiczia coccinea*, *Wuonymus japonicus*, *Wuphorbia milii*, *Zantedeschia hybrid* ‘Florex Gold’, *Zinnia elegans* [433], *Zinnia elegans* ‘Dreamland Coral’, *Zinnia elegans* ‘Dreamland Red’, and ‘Dreamland Yellow ‘ [433].
Lutein epoxide	*Aglaonema commutatum* [366], *Anthemis tinctoria*, *Calendula officinalis,* ‘Alice Orange’ and ‘Alice Yellow’ [5], *Capsicum annuum* [366], *Chrysanthemum spp*, *Chrysanthemum morifolium*, Multiplora chrysanthemum, Sunny Orange *Chrysanthemum*, *Chrysanthemum morifolium,* ‘Sunny Orange’ [5], *Convolvulus scammonia*, *Cuphea hyssopifolia* [366], *Gazania* spp. ‘Daybreak Orange’ [5], *Guzmania hybrid* [366], *Diplotaxis tenuifolia*, *Gazania* spp. ‘Day Break Yellow’, *Hemerocallis disticha*, *Hibiscus rosa-sinensis*, *Hibiscus syriacus*, *Impatiens walleriana*, *Inulina helenium* [427], *Lantana camara* [366], *Malvaviscus arboreus*, *Mentha × piperita*, *Mentha suaveolens* [366], *Narcissus poeticus* ‘Scarlet Elegance’, *Narcissus pseudonarcissus* ‘King Alfred’, *Ocimun basillicum*, *Phalaenopsis Aphrodite* [366], *Pterocaya stenoptera*, *Russelia equisetiformis* [366], *Scaevola aemula*, *Solanum laxum* [366], *Solanum lycopersicum*, *Solanum rantonnetii* [366], *Taraxacum officinalis*, *Tecoma capensis*, and *Tulipa* sp. ‘Golden Harvest’.
Lycopene	*Calendula officinalis* ‘Alice Orange’ and ‘Alice Yellow’ [5], *Begonia cavaleriei*, *Dimorphotheca aurantiaca*, *Gardenia jasminoide*, *Gazania* spp. [5], *Gazania rigens*, *Osteospermum ecklonis* [5], *Rose* ‘American Pillar’, *Rose canina*, *Rose moyesii*, *Rose pomífera*, *Rose rubrijoli*, *Rose rubiginosa*, and *Tagetes erecta.*
Neoxanthin	*Allamanda cathartica*, *Brassica napus* [5], *Capparis spinosa*, *Cosmos bipinnatus*, *Cyclamen hederifolium*, *Cyclamen mirabile*, *Cyclamen persicum* [438], *Eschscholzia California*, *Eustoma*, *Gentiana lutea* [5], *Gerbera jamesonii*, *Hemerocallis disticha* [5], *Hypericum perforatum*, *Ipomoea* sp., *Ipomoea nil*, *Ipomoea obscura*, *Lilium tigrinum* ‘Red Night’, *Lotus japonicus* [5], *Melampodium divaricatum*, *Mimulus lewisii*, *Mimulus cupreus*, *Mimulus cardianlis*, *Mimulus verbenaceus*, *Mimulus bigelovii*, *Mimulus bicolour*, *Mimulus torreyvi*, *Mimulus guttatus*, *Mimulus kelloggii*, *Mimulus jungermannioides* [428], *Nelumbo lutea* ‘American Yellow’, *Oncidium* ‘Gower Ramsey’, *Pyrostegia venusta*, wild, old-gold [5], tangerine and yellow *Solanum lycopersicum*, *Tabebuia chrysantha* [5], *Tagetes erecta* ‘Orange Isis’, *Theretia peruviana*, *Zinnia elegans* [433], *Zinnia elegans* ‘Dreamland Red’, and ‘Dreamland Yellow’ [433].
Phytoene	*Begonia × semperflorens*, *Dianthus chinensis*, *Dimorphotheca aurantiaca*, *Gardenia jasminides*, *Gazania rigens*, *Guzmania hybrid* [366], *Hieracium aurantiacum*, *Impatiens balsamina*, *Lagerstroemia indica*, *Lantana camara* yellow, red, and white [366], *Lavandula angustifolia* [366], *Magnolia grandiflora*, *Osteospermun fruticosum*, *Punica granatum*, *Rosa × hybrida,* pink and white [366], tangerine *Solanum lycopersicum*, *Solanum rantonnetii* [366], *Tagetes erecta* yellow and orange [430], *Tagetes patula* [130], *Trifolium cernuum*, *Zinnia elegans* [433], *Zinnia elegans* ‘Dreamland Red’, and ‘Dreamland Yellow’ [433].
Violaxanthin	*Agastache foeniculum*, *Agastache rugosa* [425], *Aglaonema commutatum* [366], *Allamanda cathartica*, *Allium schoenoprasum*, *Alyssum montanum* [366], *Anthemis tinctoria*, *Aphelandra squarrosa*, *Bougainvillea spectabilis*, *Brassica napus*, *Camelia chrysantha* [426], yellow and orange *Canna indica*, *Capparis spinosa*, *Capsicum annuum* [366], *Celosia cristata* [35], Sunny Orange *Chrysanthemum*, Multiflora chrysanthemum, *Chrysanthemum morifolium,* ‘Sunny Orange’ [5], *Cosmos bipinnatus*, *Cyclamen hederifolium*, *Cyclamen mirabile*, *Cyclamen persicum* [438], *Diplotaxis tenuifolia*, *Drymonia* spp., *Drymonia affinis* [366], *Eustoma grandiflorum* ‘Azuma-no-yuki’ and ‘Halley Lime’, *Dianthus caryophyllus*, *Gentiana lutea*, *Gazania* spp. ‘Daybreak Orange’, *Gazania* spp. ‘Day Break Yellow’, [5] *Helianthus annuus* ‘Sunrich Orange’ [5], *Hemerocallis disticha*, *Hypericum perforatum*, *Ipomea* sp., *Ipomea nil*, *Ipomoea obscura* [5], *Justicia aurea*, red *Lantana camara* [366], *Lilium* spp., *Lotus japonicus*, yellow *Lathyrus aphaca*, *Lilium* spp. ‘Conneticut King’ [426], *Lilium* spp. ‘Montreux’, *Melampodium divaricatum*, *Mentha × piperita*, *Mentha suaveolens* [366], *Mimulus lewisii*, *Mimulus cupreus*, *Minulus cardinalis*, *Mimulus whitneyi*, *Mimulus verbenaceus*, *Mimulus bigelovii*, *Mimulus bicolor*, *Mimulus torreyl*, *Mimulus guttatus*, *Mimulus jungermaniodes* [428], *Nelumbo lutea* ‘American Yellow’, *Nicotiana glauca* [5], *Ocimun basilicum* [366], *Omcidium* ‘Gower Ramsey’, *Plantago major*, *Pterocaya stenoptera*, *Pyrostegia venusta* [5], *Senna papillosa* [366], yellow *Solanum lycopersicum* [5], wild, old-gold, and tangerine *Rosa hybrida,* ‘Star of Persia’, *Scaevola aemula*, *Solanum lycopersicum*, *Solanum rantonnetii* [366], *Spathiphyllum montanum* [366], *Tabebuia chrysantha* [5], *Tagetes erecta* ‘Orange Isis’, *Gerbera jamesonii*, *Tagetes patula* ‘Safari Tangerine’ [5], *Theretia peruviana*, *Thumbergia alata*, *Tropaeolum majus*, *Verbena × hybrida* [366], *Viola tricolor*, *Viola wittrockiana* [307], Zinnia elegans [5], *Warszewiczia coccinea*, *Zinnia elegans* ‘Dreamland Coral’, ‘Dreamland Red’, and ‘Dreamland Yellow’ [433].
Zeaxanthin	*Agastache foeniculum*, *Agastache rugosa* [425], *Allamanda cathartica* [5], *Antirrhinum majus* ‘Snapdragon’ [307], *Brassica oleraceae*, *Canna indica* [5], *Cosmos bipinnatus*, *Cucurbita maxima*, *Crocus sativus*, *Cucumis sativus* var. Anguria, twenty-three *Dendranthema grandiflorum* [110], *Dianthus caryophyllus*, *Eustoma* [5], *Gaillardia × grandiflora*, *Gazania* spp. ‘Daybreak Orange’, *Gazania* spp. ‘Day Break Yellow [5]‘, *Gazania × hybrida*, *Gentiana lutea* [5], *Gerbera jamesonii*, *Helianthus annuus,* ‘Sunrich Orange’, *Hemerocallis disticha* [5], *Hibiscus rosa-sinensis*, *Hypericum perforatum*, *Ipomoea* sp. [5], *Ipomoea nil*, *Ipomoea obscura* [5], *Lantana camara* [366], *Lycium barbarum*, *Lycoris radiata*, *Melampodium divaricatum*, *Momordica charantia*, *Narcissus poeticus* ‘Scarlet Elegance’, *Narcissus pseudonarcissus* ‘King Alfred’ [5], *Nicotiana tabacum* [432], *Petunia × hybrida*, *Pyrostegia venusta* [5], *Rhododendron mole* [432], *Rose moyesii*, *Rose pomífera*, *Sandersonia aurantiaca* [5], *Senecio scanders*, high-pigment 3 (hp3), *Solanum lycopersicum*, *Tabebuia chrysantha* [5], *Tagetes erecta* [430], *Tagetes patula* ‘Safari Tangerine’, *Theretia peruviana*, *Tropaeolum majus* [5], *Viola witrockiana* ‘Pansy’ [307], *Zinnia elegans* [433], *Zinnia elegans* ‘Dreamland Coral’ [5], ‘Dreamland Red’, and ‘Dreamland Yellow’ [433].

**Table 4:** Some common phenolics present in flowers.

Phenolics	Edible Flowers
Chlorogenic acid	*Alyssum montanum*, *Anthemis tinctoria*, *Calendula officinalis* [101], *Campanula portenschlagiana*, *Carica papaya*, *Ceiba speciosa* [279], *Celosia cristata* [214], *Dahlia anemome*, *Ethlingera elatior*, *Fragaria × ananassa*, *Gossypium arboretum* [366], *Helianthus annuus* [456], *Heliotropium peruvianum* [366], *Ixora parviflora*, *Lycoris radiata*, *Malus* ‘Royalty’ [457], *Malus micromahus* ‘Makino’, *Malus* ‘Pink spire’, *Malus* ‘Sparkler’, *Malus* ‘Strawberry Parfait’, *Nerium oleander*, *Pelargonium peltatum*, *Pterocarya stenoptera* [366], *Rosa hybrida* ‘Scarlet’, *Saccharum edule*, *Solanum laxum*, *Tecoma stans* [214], *Trachelospermum jasminoides* [366].
Caffeic acid	*Calendula officinalis* [101], *Carica papaya*, *Celosia cristata* [214], *Convolvulus althaeoides*, *Ethlingera elatior*, *Guzmania hybrid* [366], *Helianthus annuus* [456], *Hydrangea petiolaris*, *Ixora javanica*, *Lavandula angustifolia*, *Lycoris radiata*, *Ocimun basilicum*, *Pelargonium × hortorum*, *Pelargonium peltatum* [366], *Portulaca oleracea* [214], *Saccharum edule*, *Salvia splendens*, *Trachelospermum jasminoides* [366].
Ferulic acid	*Carica papaya*, *Catharanthus roseus*, *Dahlia anemone*, *Ethlingera elatior*, *Gardenia jasminoide* [366], *Helianthus annnuus* [456], *Ixora javanica*, *Lycoris radiata*, *Nerium oleander*, *Saccharum edule* [366].
Quercetrin	*Aglaonema commutatum*, *Aloysia citrodora*, *Alstroemeria aurea*, *Anthurium andraeanum*, *Begonia argentea*, *Begonia cavaleriei*, *Begonia × semperflorens*, *Bougainvillea spectabilis*, *Cannabis sativa*, *Capsicum annuum*, *Celosia cristata*, *Centaurea sonchifolia*, *Citrullus lanatus*, *Coriandrum sativum*, *Dahlia anemone*, *Dianthus chinensis*, *Diplotasis tenuifolia*, *Escallonia rubra*, *Euonymus japonicus*, *Euphorbia milii*, *Eustoma grandiflorum*, *Fallopia aubertii*, *Fragaria ananassa*, *Fuchsia magellanica*, *Gossypium arboretum*, *Gypsophila paniculate*, *Hibiscus syriacus* [366], *Hypericum cardonae*, *Hypericum carinosum*, *Hypericum cuatrecasii*, *Hypericum garciae*, *Hypericum humboldtianum*, *Hypericum laricifolium*, and *Hypericum myricarifolium* [458], *Impatiens balsamina*, *Lathyrus aphaca*, *Limonium sinuatum*, *Mentha suaveolens*, *Nerium oleander*, *Pelargonium × domesticum*, *Pelargonium × hortorum*, *Petunia × hybrida*, *Phalaenopsis aphrodite*, *Plantago major*, *Plumbajo capensis*, *Pterocaya stenoptera*, *Rosa × hybrida* [436], *Rhododendrom simsii*, *Saponaria officinalis*, *Senna alexandrina*, *Sophora japonica*, *Tecoma capensis*, *Trifolium cernuum*, *Verbena × hybrida* [366], *Zinnia elegans* [214].
Quercetin	*Aglaonema commutatum*, *Begonia argentea*, *Bunias orientalis*, *Calendula officinalis*, *Catharanthus roseus*, *Celosia cristata*, *Dianthus chinensis*, *Diplotaxis tenuifolia*, *Escallonia rubra* [366], *Eruca sativa*, *Eruca* ssp., *Hypericum cardonae*, *Hypericum carinosum*, *Hypericum cuatrecasii*, *Hypericum garciae*, *Hypericum humboldtianum*, *Hypericum laricifolium*, *Hypericum myricarifolium* [458], *Ixora arboreoa*, *Ixora parviflora*, *Petunia × hybrida*, *Plumbago capensis*, *Rosa hybrida* [366], *Spilanthes oleracea*, *Verbena × hybrida* [214,366].
Galic acid	*Begonia cavaleriei*, *Begonia × semperflorens*, *Celosia cristata*, *Coriandrum sativum*, *Dahlia anemone*, *Dianthus chinensis*, *Euonymus japonicus*, *Euphorbia milii*, *Fallopia aubertii*, *Fuchsia magellanica*, *Gossypium arboreum*, *Heliotropium peruvianum* [366], *Hypericum cardonae*, *Hypericum carinosum*, *Hypericum cuatrecasii*, *Hypericum garciae*, *Hypericum humboldtianum*, *Hypericum laricifolium*, *Hypericum myricarifolium* [458], *Ixora finlaysoniana*, *Kalanchoe blossfeldiana*, *Limonium sinuatum*, *Lycoris radiata*, *Nerium oleander*, *Pelargonium × domesticum*, *Pelargonium × hortorum*, *Pelargonium peltatum*, *Phalaenopsis aphrodite*, *Pterocarya stenoptera*, *Portulaca oleracea*, *Rosa hybrida* [366], and *Trachelospermum jasminoides* [214].
Hydroxybenzoic acid	*Begonia × semperflorens*, *Celosia cristata*, *Chlorophytum comosum*, *Coriandrum sativum*, *Dahlia coccinea*, *Gossypium arboreum*, *Hypericum cardonae*, *Hypericum carinosum*, *Hypericum cuatrecasii*, *Hypericum garciae*, *Hypericum humboldtianum*, *Hypericum laricifolium*, *Hypericum myricarifolium* [458], *Ixora coccinea*, *Lycoris radiata*, *Mirabilis jalapa* [366], *Portulaca oleracea* [214], *Scaevola aemula*, *Solanum lycopersicum*, *Solanum rantonnetti*, *Trachelospermum jasminoides* [366].
Cumaric acid	*Agapanthus africanus*, *Agastache anisata*, *Allium schoenoprasum* [369], *Begonia cavalerier*, *Begonia × semperflorens*, *Celosia cristata*, *Campanula carpatica*, *Campanula portenschalagiana*, *Campanula shetleri*, *Canna indica*, *Catharanthus roseus*, *Cichorium intybus*, *Coriandrum sativum*, *Cucurbita maxima*, *Dahlia anemone*, *Dianthus caryophyllus*, *Euonymus japonicus*, *Euphorbia milii*, *Fuchsia magellanica*, *Gaillardia × grandiflora*, *Gazania × hybrida*, *Gladiolus communis*, *Heliotropium peruvianum*, *Jasminum sambac*, *Lantana camara*, *Lycoris radiata*, *Magnolia grandiflora*, *Mattiola incana*, *Nerium oleander*, *Osteospermun fructicosum*, *Passiflora × belotti*, *Pelargonium × domesticum*, *Pelargonium × hortorum*, *Pelargonium peltatum*, *Petunia × hybrida*, *Polygala vulgaris*, *Portulaca oleracea*, *Rosa hybrida*, *Russelia equisetiformis* [366], *Taraxacum officinale* [214], *Verbena × hybrida*, and *Vitex agnus-castus* [366].
Kaempferol	Purple amaranth, *Antirrhinum majus*, *Begonia × semperflorens*, *Bunias orientalis*, red and Orange Capuzin, *Catharanthus roseus*, *Celosia cristata* [366], *Chrysanthemum spp*, *Chrysanthemum morifolium* [111], *Colvolvulus scammonia*, *Coreopsis grandiflora*, *Dahlia anemone* [366], *Dahlia pinnata*, *Eruca sativa*, *Eruca* ssp., *Hypericum cardonae*, *Hypericum carinosum*, *Hypericum cuatrecasii*, *Hypericum garciae*, *Hypericum humboldtianum*, *Hypericum laricifolium*, *Hypericum myricarifolium* [458], *Impatiens walleriana*, *Ixora coccinea*, *Limonium sinuatum*, *Malvaviscus arboreus* [366], *Punica granatum* [406], *Rosa hybrida* [366], *Spathiphyllum montanum*, and *Tagetes patula* [214].
Anthocyanins	*Bidens ferulifolia*, *Capsicum annuum*, *Catharanthus roseus* [214], twenty-three *Dendranthema grandiflorum*, *Ixora chinensis*, *Lycoris radiata*, *Malus* ‘Royalty’, *Malus micromahus* ‘Makino’, *Malus* ‘Pink spire’, *Malus* ‘Sparkler’, *Malus* ‘Strawberry Parfait’ [279], *Punica granatum* [406], *Rosa* spp. ‘Mister Lincoln’, ‘Papa Meilland’, *Rosa rugosa* ‘Veilchenblau’, ‘Better Times’, ‘María Callas’, ‘Queen Elizabeth’, *Spilanthes oleracea*, *Solanum melongena*, *Tropaeolum majus*, *Tagetes erecta*, *JasminePrimu majus* [459], *Zantedeschia hybrid* ‘Albomaculata’, ‘Black Magic’, ‘Florex Gold’, ‘Mango’, ‘Majestic Red’, ‘Chianti’, ‘Treasure’, ‘Pink’, and ‘Persuasion’ [460].

## Data Availability

The datasets generated for this study are available on request to the corresponding author.
